# Constraints on multi-item working memory access: performance costs and retrieval dynamics

**DOI:** 10.3389/fpsyg.2025.1558689

**Published:** 2025-04-09

**Authors:** Chen Tiferet-Dweck, Abigail Keegan, Kerstin Unger

**Affiliations:** ^1^Department of Psychology, Queens College, City University of New York, New York, NY, United States; ^2^The Graduate Center, City University of New York, New York, NY, United States

**Keywords:** multi-item working memory access, output gating, parallel vs. serial working memory retrieval, retrocuing, working memory

## Abstract

To support goal-directed behavior, working memory (WM) must flexibly access relevant information. While the mechanisms underlying single-item WM access are comparatively well-studied, less is known about the principles governing multi-item access. Some studies have suggested that dual-item retrieval can be as efficient as single-item access, but it remains unclear whether this reflects reduced inhibitory demands or truly parallel, cost-free retrieval. In Experiment 1, we manipulated the number of relevant vs. irrelevant items in a pre-and retro-cuing WM task. The rationale was that if reduced inhibitory demands benefit multi-item access, then having fewer irrelevant items to suppress would enhance performance. Instead, we found that selecting two out of three items was slower and less accurate than selecting one, arguing against the idea that diminished inhibition underlies multi-item retrieval efficiency. Experiments 2a and 2b further probed retrieval efficiency using a modified dual-access paradigm that leveraged object repetition benefits. By including a control condition to prevent temporal associations between repeated targets and non-targets, we observed that repetition benefits for each item were additive—consistent with serial or limited parallel retrieval—rather than overadditive, which would be expected under fully parallel, cost-free retrieval. These findings clarify key limitations of multi-item WM, with important implications for complex tasks such as language comprehension, decision-making, and problem solving.

## Introduction

Working memory (WM) is a system responsible for temporarily holding and actively manipulating information to support ongoing mental operations and motor tasks. Given the strict capacity limitations of WM, a complete account of its function must address how its contents are controlled. A critical aspect of this control is the ability to selectively prioritize specific memory representations as they become behaviorally relevant (Buschman and Miller, 2022; Chatham and Badre, 2015; Myers, 2022). Selective WM output control is essential in many complex tasks—such as language comprehension, reasoning, and learning—where a uniform, simultaneous biasing influence of all maintained contents would often result in interference or even behavioral conflict. Consider, for example, a chess player with the White pieces who, while scanning the board, encodes several potential defensive and offensive strategies into WM. These possibilities might include developing the knight to g5, advancing a pawn forward on the king side, or repositioning the queen to launch an attack against Black’s king. All these candidate moves are maintained simultaneously. Now, suppose Black makes a pawn move intended to protect the king but inadvertently creates an opportunity for White to execute the contemplated move of the queen to h5. This triggers a forced sequence culminating in checkmate, rendering the other maintained strategies obsolete.

Importantly, it is not always a single item that needs to be prioritized; multiple contents may be required simultaneously for ongoing mental operations. This kind of multi-item “read-out” is necessary when solving math problems or integrating multiple pieces of information to draw new inferences (Gilchrist and Cowan, 2011; Oberauer, 2013; Oberauer and Bialkova, 2009). Despite considerable progress in understanding the mechanisms underlying the access to single items in WM (Buschman and Miller, 2022; Souza and Oberauer, 2016), comparatively less is known about the principles that govern multi-item access. This study aims to identify factors that determine the speed and efficiency of retrieving multiple items from WM.

## The potential role of inhibitory mechanisms in multi-item access

Several theoretical WM models propose that information is maintained in varying states of accessibility. A broad set of task-relevant representations remains activated above baseline in long-term memory, while a small subset is held in a privileged state—referred to as focus of attention—which is characterized by enhanced precision and activation. Only the contents within the focus can directly influence other cognitive processes and behavior (Cowan, 2001; Oberauer and Hein, 2012). According to this view, accessing a specific item in WM involves shifting it into the focus of attention—a process that is commonly thought to be guided by context representations (Chatham and Badre, 2015; Oberauer and Lin, 2017, 2023). Specifically, interference-based computational models of WM posit that encoding entails the formation of temporary^1^ bindings between each item and a context—whether spatial location, temporal features, or semantic attributes—that can later serve as retrieval cue to re-activate the associated item (Lewandowsky and Farrell, 2008; Oberauer and Lin, 2017, 2023). Working memory access is constrained by the strength and precision of these item-context bindings. Since both context and item representations have limited precision, a specific context may activate not only the target item but also other maintained contents to the extent that they share similar contexts or overlapping features. This can lead to retrieval failures, confusion, and slower accumulation of evidence for the target when memory is probed.

Intuitively, one might therefore expect that accessing multiple items in WM is more error prone and time-consuming due to increased competitive interactions and interference among retrieval candidates (Collins and Frank, 2013; Manohar et al., 2019; Desimone and Duncan, 1995). Contrary to this notion, several studies have shown that selecting a single item from WM can incur a greater performance cost than selecting multiple items (Chatham et al., 2014; Unger et al., 2016). These studies used a retro-cuing task in which participants first encoded two stimuli into WM and later received a retrospective cue specifying whether one or both items were relevant. Immediately afterward, a recognition test showed slower response times (RT) and higher error rates higher when focusing on a single memory item rather than both. This counterintuitive finding has been attributed to the interplay between facilitatory and inhibitory mechanisms in selective WM access (Chatham et al., 2014). In particular, neurobiologically inspired computational models propose that distinct cortico-striatal loops enhance behaviorally relevant WM content while inhibiting irrelevant content, with crosstalk between these loops potentially slowing single-item access. This notion aligns with the broader view that retrospective cues in WM tasks typically not only highlight a subset of items as relevant but also indirectly mark the remaining items as irrelevant, possibly prompting their removal from WM. Yet, previous research suggests that such removal is often incomplete—irrelevant items are typically not unbound from their contextual representations but are instead de-activated below baseline levels (Oberauer, 2018).

Importantly, the studies reporting a performance advantage for dual- over single-item WM access have two major limitations. First, they have relied on a task paradigm in which dual-item access is *global* and indiscriminate—encompassing the entire memory set—rather than selectively prioritizing a specific subset of maintained WM contents. This distinction is critical, as evidence suggests that the mechanisms supporting selective and global WM access differ (Oberauer, 2005, 2019; Oberauer and Lin, 2017). Specifically, selective WM access is believed to depend on item-context bindings to differentiate between relevant and irrelevant stimuli (Lewandowsky and Farrell, 2008; Oberauer and Lin, 2017, 2023). In contrast, global WM access merely requires an individual to remember which items are part of the current memory set, without the need to differentiate between them. In such cases, decision-making can rely solely on item memory (cf. Oberauer, 2019). Thus, a key goal of this study was to examine the potential contributions of inhibition vs. enhancement in WM access by comparing single-item access to *selective*, rather than global, dual-item access.

A second limitation of previous studies contrasting single- and multi-item retrieval is that RT and accuracy data were based on responses to a probe display, leaving it unclear to what extent these measures reflect WM selection versus the processing of the memory probes. Although the two processes are interconnected—the nature of the selection process most likely depends on the requirements of the task that prompts it—the memory test involves additional components such as comparing the probe to the maintained target representation(s), visual search demands, and episodic retrieval processes triggered by the probe itself. Indeed, prior research suggests that the selection of the relevant WM representation and its subsequent comparison with a memory probe are dissociable processes that do not fully overlap (Shepherdson et al., 2018). Another objective of the present study, therefore, was to measure the speed of WM selection independently from subsequent probe responses.

## Is multi-item access serial or parallel?

**Figure 1:**
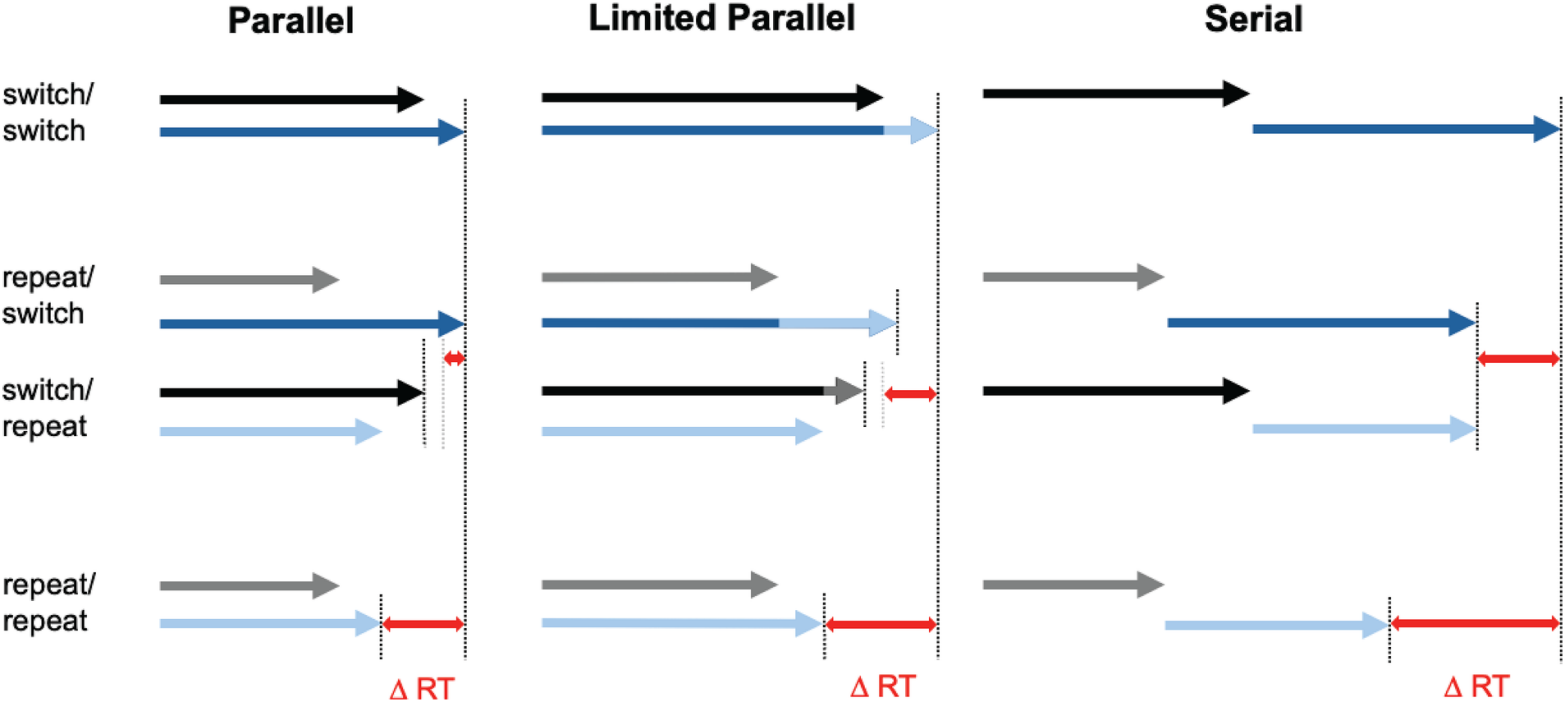
Schematic illustration of the effects of item repetition on dual-access speed in parallel (left) vs. serial (right) retrieval. Each arrow illustrates the time required to access one of the two target items in the dual-access test. Blue arrows correspond to the target item with slower retrieval time, while black/gray arrows represent the target with faster retrieval. Darker colors (black and dark blue) indicate baseline retrieval times without repetition benefits (switch), while lighter colors show accelerated access due to repeated access to the same item (repeat). Red double-headed arrows represent the overall repetition benefit (DRT) in the dual-access test for repeating a single target (repeat/switch, switch/repeat) or both targets (repeat/repeat) compared to repeating neither of the targets (switch/switch). In unrestricted parallel access model (left panel), the RT benefit of repeating a single target (repeat/switch and switch/repeat) is very small because the slower target constrains overall retrieval time. By contrast, the benefit of repeating both targets (repeat/repeat) is much larger, resulting in an overadditive effect—that is, the RT benefit exceeds the sum of the individual benefits of repeating a single target. The distinction between switch/repeat and repeat/switch conditions is shown for illustrative purposes only, to highlight the differential effects of repeating the faster vs. slower target in the parallel-access model. In this model, repeating the faster target does not affect overall retrieval time, while repeating the slower target results in a small benefit. In the actual experiment, these two cases were not meaningfully separable. Consequently, observed RTs in the repeat/switch condition reflect the average benefit across both cases. In the serial access model (right panel), the repetition benefit for the repeat/repeat condition is additive, approximately equal to the sum of the individual benefits of repeating a single target. Note that a similar performance pattern could emerge when two items are retrieved *partially* in parallel—albeit at a slower pace than single-item retrieval—resulting in substantial RT slowing for dual-item access (middle panel). In this scenario, the benefit of repeating one target twice may arise from an acceleration in the retrieval process of the non-repeated target after the repeated target has been accessed. The initial phase of simultaneous retrieval may act as a bottleneck; once it is overcome, the retrieval of the second target can proceed at the faster pace. This increase in speed after competition of retrieval of the first item is indicated by lighter colors. Figure adapted from Oberauer (2013).

Another line of evidence suggesting that multi-item access in WM can sometimes be as efficient as single-item access comes from research on whether multiple items can be retrieved into the focus of attention simultaneously (parallel retrieval) or only sequentially (one item at a time; Gilchrist and Cowan, 2011; Oberauer, 2013; Oberauer and Bialkova, 2009; Oberauer and Kliegl, 2004). This question resonates with the broader debate on parallel vs. serial processing in dual-task situations, where performance costs have been attributed to a central processing bottleneck, crosstalk between shared processing streams, strategic considerations, or a combination of these factors (for review, see Fischer and Pleskow, 2015; Hirsch and Koch, 2024). There is a broad consensus that the relative contributions of seriality and parallelism in dual-task processing vary depending on various factors, such as the amount of task practice, reward expectations, or the specific conditions under which the tasks are performed (Fischer and Plessow, 2015; Hommel, 2020). Although memory retrieval, particularly when it engages cognitive control, is often viewed as constrained by parallel-processing limitations, prior work indicates that two WM items can be retrieved in parallel without incurring additional cost—even in the absence of extensive task practice (Oberauer, 2013). Rather than comparing single- vs. multi-item access, these studies have used a dual-access paradigm that capitalizes on a well-established finding: context-based retrieval of a memory item is facilitated when that same item has been retrieved immediately before (object repetition benefit or object switch cost; e.g., Oberauer, 2003; Verhaeghen et al., 2004). Several factors may contribute to this facilitation, among them cue priming, strengthened associations between items and retrieval cues, and heightened activation of recently accessed item(s) (Oberauer and Hein, 2012). Crucially, if multiple items are accessed serially—meaning the retrieval of the next item only begins after the previous one is fully retrieved—the overall RT depends on the retrieval speed of each individual item. In this case, if two items need to be retrieved and one of them was accessed previously, the gain for the repeated item should reduce the overall RT. If both items were accessed in the preceding trial, the RT benefit should equal the sum of the individual repetition benefits for each item (see Figure 1 for an illustration).

Notably, a similar performance pattern could emerge when two items are retrieved *partially* in parallel—albeit at a slower pace than single-item retrieval—resulting in substantial RT slowing for dual-item access. In this scenario, the benefit of repeating one target twice may arise from an acceleration in the retrieval process of the non-repeated target after the repeated target has been accessed. The initial phase of simultaneous retrieval may act as a bottleneck; once it is overcome, the retrieval of the second target can proceed at the faster pace characteristic for single-item access—akin to “taking the foot off the brake.” Consequently, the less overlap there is between the retrieval processes for the two targets, the more quickly the slower process will conclude. Such limited parallel access is consistent with capacity sharing and crosstalk models of dual-task performance (e.g., Tombu and Jolicoeur, 2002; Lien and Proctor, 2002).

In contrast, if retrieval of two items were entirely parallel and unrestricted, the number of repetitions of one target—and the resulting increase in its retrieval speed—would have no impact on dual-access RT. The time required to access the second, unrepeated target would remain unchanged, ultimately limiting response speed. However, when both items are repeated, faster access to each item should result in a much greater RT benefit. Therefore, serial/limited parallel vs. fully parallel retrieval would produce distinct performance patterns: in serial/limited parallel retrieval, the RT benefit of repeating both items would be additive, while in truly parallel, unrestricted retrieval, the benefit would be overadditive (Figure 1). Importantly, in the dual-access paradigm only the to-be-selected items repeat across consecutive retrieval attempts—not the corresponding responses. Thus, any RT differences could be attributed specifically to facilitated cue-based access to the relevant items, rather than to differences in response selection or execution.

In one study using this approach, participants were shown a sequence of four colored digits and then performed a series of arithmetic operations, each involving two of the presented digits (dual access; Oberauer and Bialkova, 2009). Color cues indicated which memory items were relevant for each operation. From one operation to the next, either both, one, or none of the relevant items would repeat, while the operation itself would change. The results revealed an overadditive benefit when both digits were reused compared to when only one of the required digits was reused, which has been interpreted as evidence for cost-free, parallel retrieval (Oberauer, 2013; but see Gilchrist and Cowan, 2011). However, there is a caveat to this conclusion: Each time two items were accessed for a new operation, they were likely re-encoded into memory (Yonelinas et al., 2019). This re-encoding may not only strengthen the association between each item and its color context but also create an episodic memory trace that binds both items, their spatial cues, and the current temporal context into a unified event (Beukers et al., 2021; Karpicke et al., 2014).^2^ Although temporal context gradually shifts over time, it is generally thought to remain sufficiently stable within a trial to effectively cue retrieval of the most recent event representation. As a result, the temporal context of the current memory test, combined with any repeated color cue, would preferentially activate the two digits from the preceding operation. This is beneficial when both repeated items are also used in the current operation. In such cases, temporal context and color cues work together to facilitate retrieval of the relevant item pair, speeding up dual-item access. However, if only one item is reused, the episodic memory trace may become less helpful or even detrimental, as temporal context, the repeated color cue, and activation of the repeated target can also trigger retrieval of the associated non-target. In this situation, participants must distinguish between the relevant and irrelevant repeated item, a process that depends on the unique binding of each item to its color context. Consequently, the repetition benefit conferred by the temporal context is eliminated. Finally, when neither item is repeated from the previous operation, the event memory trace is only reactivated by temporal context, not by the color cues, reducing the likelihood of retrieving the previous digits. This asymmetry may explain why Oberauer and Bialkova (2009) observed an overadditive RT benefit when both items were repeated compared to when only one was repeated. In their study, the authors concluded that the two repeated items were chunked into a single unit that could only be accessed and used as a whole—a concept somewhat similar to the rationale we have described here. However, in their account, the items are combined into an ad-hoc chunk *before* entering the focus of attention, with repetition benefits arising from carrying that chunk over between retrieval attempts. In contrast, we propose that the two items are accessed individually but become associated as a *result* of being integrated into the same episodic event representation.

In summary, prior research on multi-item WM access has suggested a counterintuitive possibility: retrieving two items can be as efficient as, or even more efficient than, retrieving only one item. However, these findings remain inconclusive for at least two reasons. First, studies that directly compared single-item vs. dual-item access often confounded the comparison by requiring selective WM access for single items but global WM access for dual-item retrieval. Second, investigations into serial vs. parallel in dual-item retrieval have not fully accounted for temporal context effects, which may have biased the results.

## The present study

The primary goal of this study was to rigorously test whether cue-based multi-item WM access can be as efficient as single-item access. Specifically, we sought to determine whether multi-item retrieval benefits from reduced inhibitory demands, ad-hoc chunking, or truly cost-free parallel retrieval. To address these aims, we designed two sets of experiments. *Experiment 1* compared selective single-item and dual-item access to assess the roles of inhibition and enhancement in cue-based WM retrieval. If selection is slowed by the need to inhibit or deactivate irrelevant contents, this slowing should increase with the number of irrelevant items. That is, given a constant overall set size, single-item access should be slower and possibly less accurate than dual-item access. Alternatively, if cue-based selection relies primarily on one-by-one enhancement of relevant contents, performance costs should be greater for dual-item access than for single-item access. To foreshadow, the results of Experiment 1 supported this latter hypothesis, suggesting that multi-item retrieval is driven by selective enhancement rather than by inhibition and challenging previous conclusions regarding the serial vs. parallel nature of multi-item WM retrieval.

To resolve this inconsistency with prior findings, *Experiments 2a* and *2b*examined whether the overadditive benefit of repeating both targets in the dual-access paradigm (Oberauer and Bialkova, 2009)—previously interpreted as evidence for cost-free, parallel retrieval—might instead result from unintended temporal associations between repeated items. Two key modifications were introduced to the original paradigm. First, rather repeating items in pairs, which might cause them to be bound into a single episodic event, we repeated only one item at a time. Specifically, we tested the cumulative effect of two consecutive single-access tests in a subsequent dual-access test. This approach reduced the likelihood that both repeated items would be merged into one unit or chunk, with later access to one automatically triggering the retrieval of the other. Second, to further control for potential associations between repeated items, we included a novel condition in which the same target was repeated twice instead of repeating two distinct items once. If dual-item access were truly parallel and unrestricted, the benefit of repeating both targets rather than just one should remain overadditive—regardless of whether the targets are repeated sequentially or simultaneously, or whether a single target is repeated once or twice. In an unrestricted parallel system, overall response speed would be determined solely by the slower retrieval process—namely, that of the unrepeated target—so the magnitude of the repetition benefit for the repeated target would not affect overall response time (cf. Figure 1).

## Experiment 1

**Figure 2:**
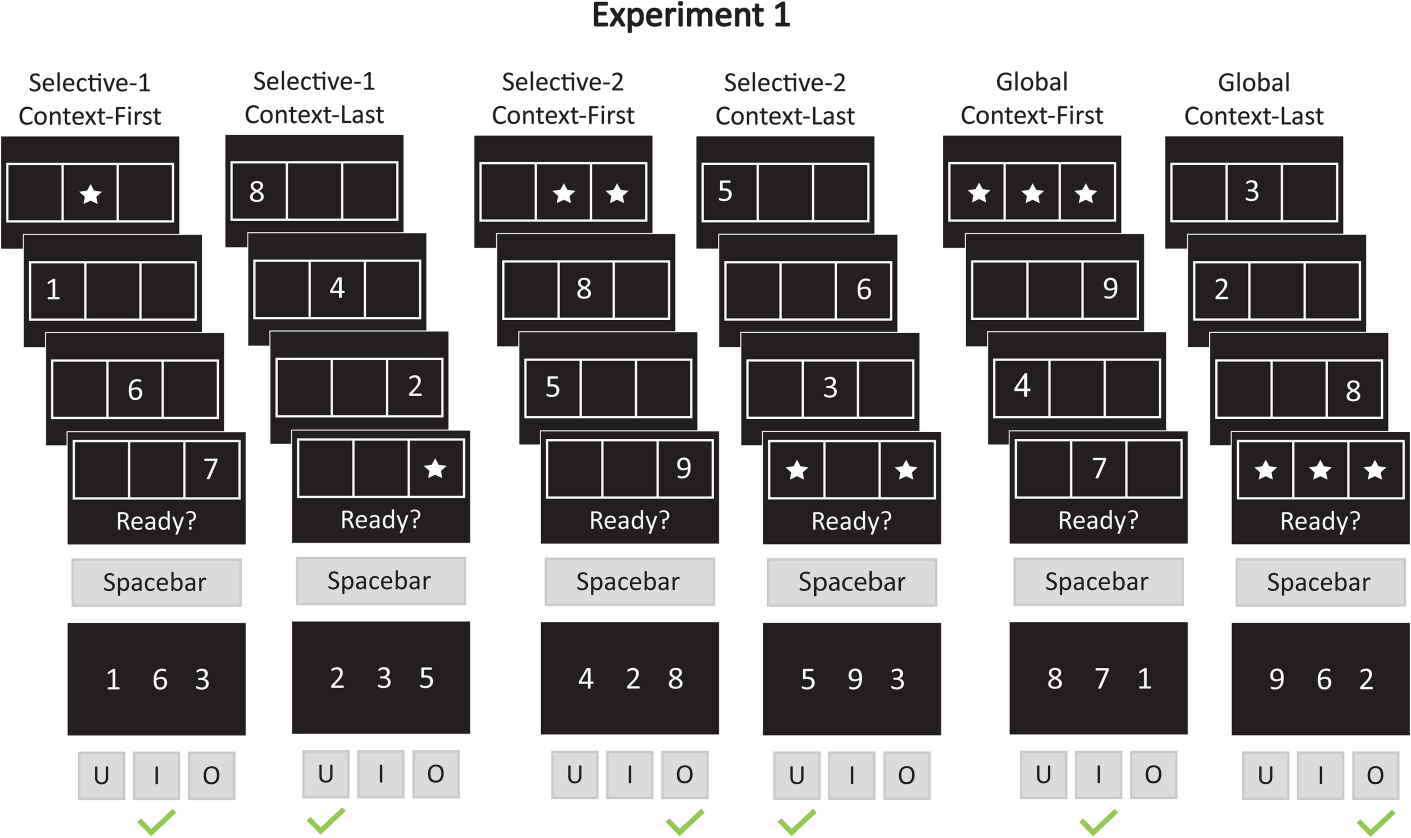
Example trials for experiment 1. In Experiment 1, participants were shown digits (1–9) in one of three locations on a horizontal grid. Context cues, represented by stars, marked the location of the relevant items. There were three cue conditions: selective-1 (one item relevant), selective-2 (two items relevant), and global (all three items relevant). The context cues appeared either at the beginning of the sequence (context-first) or at the end (context-last). Simultaneously with the final stimulus in a sequence—either the third memory item or the spatial cue—the prompt “Ready?” appeared on the screen, signaling participants to select the relevant items from memory. Participants indicated the completion of this selection step by pressing the spacebar (first response). Immediately after this response, a three-item probe display appeared on the screen. The probe display included one target (a relevant item) and two lures. Intrusion probes included one irrelevant item from the same trial (same-trial lure) and one item from a different trial (other-trial lure). Non-intrusion probes, by contrast, contained two other-trial lures. In the selective conditions (selective-1 and selective-2), probes were evenly split between intrusion and non-intrusion types (50% each). In the global condition, only non-intrusion probes were presented. Participants selected the target by pressing one of three response keys: “U,” “I,” or “O,” corresponding to the left, middle, and right positions, respectively (second response). For example, in a selective-2 context-last trial, the probe might display the target digit “5” (left position), a same-trial lure “3,” and an other-trial lure “9.” In this case, the correct response would be to press the “U” key.

In Experiment 1, we adapted a previously established pre- and retrocuing WM task (Chatham et al., 2014) to examine whether selecting multiple items from WM, while keeping the overall memory load constant, is less time-consuming and error-prone than selecting a single item. Such a performance difference would suggest that selection costs arise from the need to inhibit irrelevant items rather than from context-based enhancement of the relevant ones. Participants viewed sequences of three digits presented in distinct positions on a horizontal grid. Context cues—in the form of stars—were displayed either before (pre-cue) or after (retro-cue) the digits, highlighting one, two, or all three grid locations to indicate which items would be relevant for the forced-choice recognition test at the end of each trial (see Figure 2). When all three items were cued (*global* access), there was no need to differentiate between them. In contrast, when only one item or two items were cued (*selective* single- or dual-item access), participants had to disregard the irrelevant items. In selective pre-cue trials, participants could encode only the relevant items into WM based on the initial cue, thereby reducing memory load and enabling proactive control over WM contents. Conversely, retro-cue trials required that all three items be held in WM until the final cue specified the relevant subset, necessitating retrospective control. Our primary focus is on the retro-cue condition. Unlike earlier versions of the paradigm—which recorded responses only during the final recognition test—the present task also required participants to respond to an earlier readiness prompt. This prompt was presented simultaneously with the final stimulus in each sequence—either the context cue (in retro-cue trials) or the third digit (in pre-cue trials). Participants were instructed to press a key as soon as they had retrieved the relevant item(s), with the reaction time for this initial response capturing the speed of WM access before any probe processing occurs. Only afterward was the probe display presented, requiring participants to identify the target digit (one of the relevant items) that appeared alongside two lures.

Our *hypotheses for Experiment 1* were as follows: We predicted that probe RTs would increase as the number of relevant WM representations increases from one in selective single-item access to three in global access, reflecting a relevant-set size effect (Bunting et al., 2006; Ma et al., 2014; Oberauer et al., 2018; Risse and Oberauer, 2011; Sternberg, 1969). In contrast, readiness RTs—indicating the speed of WM retrieval—should help isolate the role of inhibitory demands. If inhibition demands increase with the number of irrelevant items, selective single-item access (with two irrelevant items) should be slower than selective dual-item access (with one irrelevant item). Alternatively, if dual-item access relies primarily on enhancing the activation of relevant items, the opposite pattern should emerge: boosting the activation of two items above their competitors—each by itself a stochastic process—should take more time than boosting just one. Similarly, if selection operates by inhibiting irrelevant items individually, error rates should be higher for single- than dual-item access, as reflected in the subsequent probe response. Conversely, if selection chiefly relies on individually enhancing relevant items, we would expect higher error rates for dual-item access.

### Methods

#### Participants

Fifty-two young adults participated in this study. To ensure adequate statistical power, we aimed for an effective sample size of approximately *n* = 40. This number was determined based on power analyses that used data from a previous, unpublished experiment (*n* = 41) to estimate effect sizes. Power estimates for all fixed effects and interactions of interest (see below section “Data Analyses”) were computed using the R package *mixedpower* (v0.1.0; Kumle et al., 2021). Participants were recruited from the crowdsourcing platform Prolific^©^ and were pre-screened for their location, age, mother tongue, and health. Only individuals who lived in the United States, were between 18 and 40 years old, native English speakers, and reported normal or corrected-to-normal vision and hearing took part in the studies. They received $12 for their participation. Four participants were excluded from data analyses due to performing at chance level in one or more of the experimental conditions (accuracy ≤ 50%). Note that this exclusion criterion was based on responses to the probe only as accuracy could not be meaningfully measured for responses to the readiness prompt. Data from another participant was discarded because their response times indicated that they did not pay sufficient attention to the task (RTs > 6 s on more than 10 trials). The final sample included 47 datasets, the mean age was 27.3 years (*SD* = 4.94), ranging from 18 to 40 years. Twenty-two participants were female, 25 were male (see Supplementary Methods for racial/ethnic information). Informed consent was obtained from all participants prior to the experiment, and they were debriefed at its conclusion. The experimental procedures were approved by the Queens College Institutional Review Board.

#### Materials, design, and procedure

##### Materials

Each memory set comprised three digits (ranging from 1 to 9), presented sequentially in one of three positions within a horizontal 3 × 1 grid (Figure 2). Stars appeared in specific grid positions, serving as higher-order context cues to indicate which digits were relevant for the recognition test at the end of each trial. During the recognition test, probes were displayed as three horizontally arranged digits—one target and two lures. All stimuli were presented in light-gray font against a black background. The digits measured about 2.5° × 2.5° and the grid extended over a visual angle of about 10.5° × 3.5°.

##### Design

The study used a 2 × 3 × 2 within-subjects design, with the following independent variables and corresponding levels: (1) *Context Position* was manipulated across two levels: context cues were shown either before (*context-first*) or after (*context-last*) the digits; (2) *Relevant Set Size* had three levels: in *selective-1* trials (single-item access), one star marked a single relevant item, in *selective-2* trials (dual-item access), two stars marked two relevant items, and in *global* trials, three stars indicated that all items were relevant; (3) *Probe Type* had two levels based on the nature of the lures: *other-trial lures* consisted of digits not used in the current trial, while *same-trial lures* were digits from the current trial that had been marked as irrelevant. Context Order and Relevant Set Size were fully crossed, whereas Trial Type and Relevant Set Size were not. This was because global trials could not include same-trial lures, as all digits in the memory set were relevant. As a result, the task involved 11 unique conditions.

Three dependent variables were assessed: *Readiness RT*, *Probe RT*, and *Probe Response Accuracy*. *Readiness RT* served as a measure of WM retrieval speed, unaffected by processing of the subsequent memory probe. Participants indicated that they had retrieved the relevant items by responding to a readiness prompt (the phrase “Ready?”) that appeared at the end of each stimulus sequence. However, this readiness response was nonspecific—the same key was pressed in each trial—and did not assess the correctness of WM retrieval, i.e., how well participants distinguished relevant from irrelevant items. Instead, retrieval accuracy was captured by *Probe Response Accuracy*, which reflected correct identification of the target in the recognition test. Finally, *Probe RT* reflected how efficiently participants used pre- and retro-cues to narrow down the set of WM representations competing for retrieval (relevant-set size effect), with faster responses expected when fewer items were relevant.

##### Procedure

All participants enrolled in the studies through Prolific^®^ and accessed the experiment via a participation link. Prior to beginning the task, they provided informed consent. Following this, they completed a brief demographic questionnaire and viewed instructional videos explaining the task procedures. The experimental task started with a short practice phase consisting of 20 trials. If a participant did not meet the performance criterion (70% correct trials), they were given the option to either exit the study or repeat the practice phase up to two times.

In each trial of the WM task, four stimuli—three digits and one context cue—were presented one at a time for 250 ms each, with fixation intervals randomly varying between 1,000 and 1,500 ms (see Figure 2, for example trials). The digits and their spatial positions were randomly selected without repetition within a trial. At the same time as the final item in each sequence—either a digit or a star display—the phrase “Ready?” appeared on the screen. This readiness prompt remained visible until participants pressed the space bar with their thumb, indicating that they had retrieved the relevant items. Immediately upon the readiness response, the probe display appeared in the center of the screen and remained for a maximum of 1,500 ms or until participants had selected the target (probe response). The probe response deadline discouraged premature responses to the preceding readiness prompt, ensuring participants completed item selection before the memory test. In the selective-1 and selective-2 conditions, 50% of the trials included non-intrusion probes containing two other-trial lures (digits not presented in the current trial), while the other 50% included intrusion probes containing one same-trial lure (a digit from the current trial that was irrelevant), paired with an other-trial lure. In contrast, the global condition, where all three items were relevant, probes always contained two other-trial lures. The inclusion of same-trial lures in the selective conditions discouraged indiscriminate retention of all three items, regardless of the context cue (cf. Oberauer, 2005). For conditions with multiple relevant items (selective-2, global), the target was randomly selected from the relevant set. In the selective-1 condition, the same-trial lure was randomly chosen among the two irrelevant items, while in the selective-2 condition, the single irrelevant item automatically served as the same-trial lure. Other-trial lures, in all cases, were randomly drawn from the pool of unused digits. The positions of the target and lures within the probe array were randomized. Participants selected the target stimulus by pressing the “U” (leftmost stimulus), “I” (middle stimulus), or “O” (rightmost stimulus) keys on the keyboard, using their right hand’s index, middle, and ring fingers, respectively. Participants were told to keep their fingers positioned over the response keys. Throughout all fixation periods, an empty grid remained on the screen. Trials were separated by a 1,000–1,250 ms fixation interval.

The order of the experimental conditions was randomized, and stars were positioned such that each grid location was marked as relevant equally often over the course of the task. In pre-cue trials, i.e., when the higher-order context cue appeared before the lower-order items (*context-first*), participants could selectively focus on updating only the relevant subset of items into WM, disregarding the irrelevant ones. This strategy effectively reduced WM load in the selective-1 and selective-2 conditions compared to the global condition, where all items were relevant. Conversely, in retro-cue trials where the context cue appeared after the lower-order items (*context-last*), participants were required to maintain all three items and selectively access only the relevant representations upon cue presentation. The task comprised six blocks of 36 trials each, resulting in a total of 216 trials. Blocks were separated by self-paced breaks. Upon completing the task, participants were provided with a downloadable debriefing document. The entire session lasted approximately 45 min.

The task was created using Labvanced^®^ online experiment software (Finger et al., 2017). Participants completed the experiments on their personal laptops or desktop computers, which had to be equipped with internal or external speakers and a US keyboard. The minimum required physical screen size was 13 inches, with a minimum resolution of 800 × 600 pixels.

#### Data analysis

##### Data preprocessing and general analysis approach

All analyses for this and the following experiments were performed using R version 4.2.0 (R Core Team, 2022). In a first step, the first five trials of the task as well as trials with response latencies under 200 ms or more than 8 s (readiness RT) were excluded. In a second step, trials with RTs more three *SD* above the individual mean for each condition were removed (readiness or probe RTs). Only correct trials were included in the RT analyses. Readiness RTs, probe RTs, and probe response accuracy were fitted on a single-trial level using (generalized) linear mixed models as implemented in the R package *lme4* (v1.1–35.3; Bates et al., 2015). The generalized mixed-effects models included a logit link function. Maximum likelihood estimation was used to fit the model parameters. Degrees of freedom were approximated using the Satterthwaite method (R package *lmerTest,* v3.1–3; Kuznetsova et al., 2017). All models were initially set up to include participant-wise random intercepts and random slopes for the fixed effects. In case the data did not support the random slopes for each within-subjects main effect, the random-effects structure was simplified following the model selection criteria suggested by Bates et al. (2015) and Matuschek et al. (2017). Successive difference contrasts were used for all predictors so that the coefficients were the difference in means between the two factor consecutive factor levels, i.e., level 2 minus level 1, level 3 minus level 2. Standardized model parameters are reported and were calculated using the *parameters* package (Lüdecke et al., 2020). The function *confint* from the *stats* package was used to calculate likelihood profile confidence intervals for all parameter estimates. Significant interactions were followed up with pairwise post-hoc comparisons using the *emmeans* package (v1.10.1, Lenth, 2024). Šidák correction was used to control the family-wise error rate.

##### Readiness RT model

The model for the readiness RTs included fixed effects for context position (context-first, context-last) and relevant set size (global, selective-1, selective-2).

##### Probe responses models

Probe RTs and response accuracy were analyzed using two separate models, each including fixed effects for context position (context-first, context-last), relevant set size (selective-1, selective-2, global), and distractor type (other-trial lure, same-trial lure). Due to the unbalanced design—specifically, the absence of same-trial lures in the global condition—the corresponding columns were omitted from the design matrix.

### Results

#### Readiness RT

**Figure 3:**
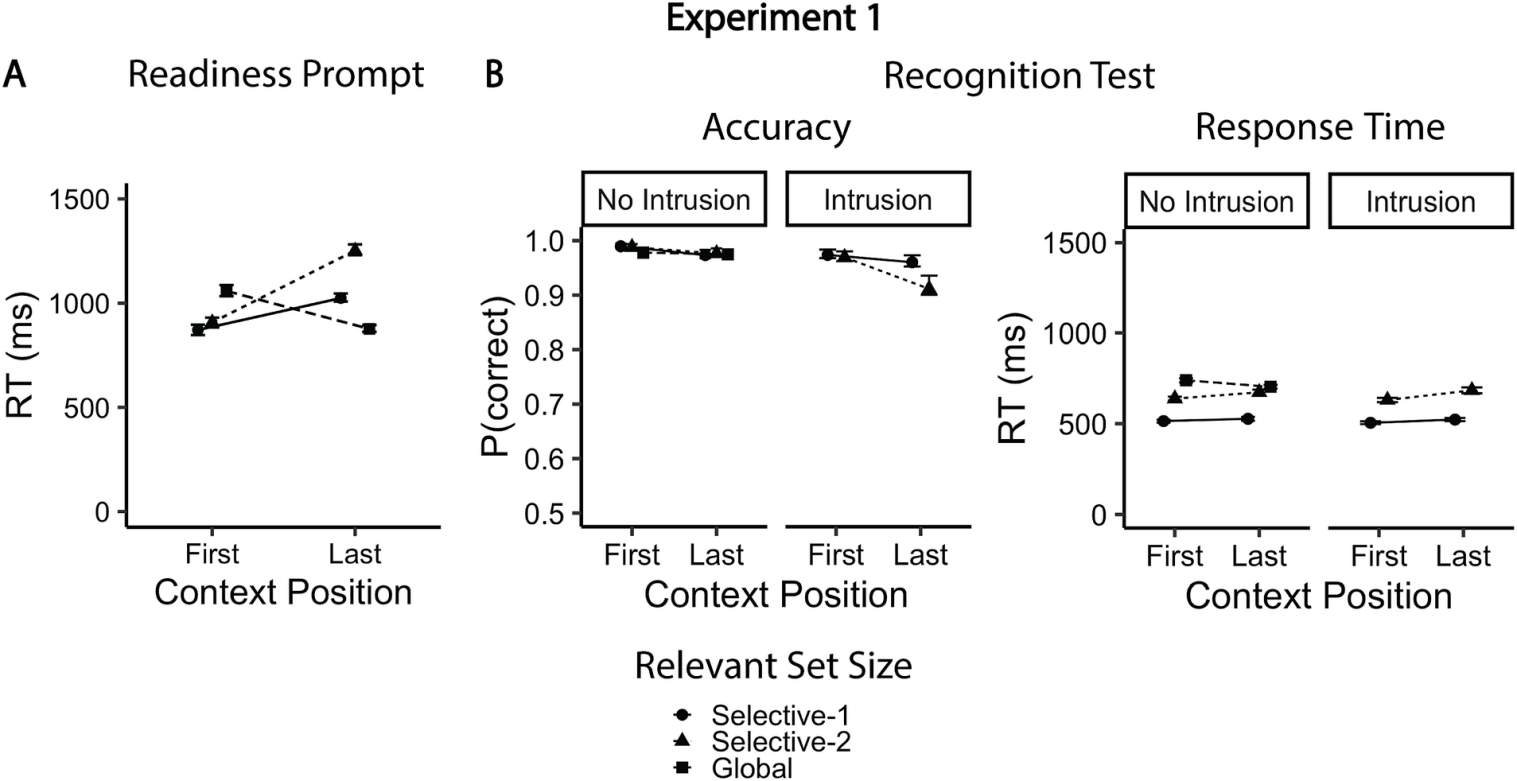
Mean accuracy rates and reaction times for experiment 1. **(A)** Mean response times to readiness prompt as a function of context position and relevant set size. **(B)** Mean accuracies and responses times for the recognition test. The global condition included only non-intrusion probes (other-trial lures). Error bars represent within-subjects confidence intervals (95%), estimated using bootstrapping (percentile-based intervals).

Response speed was faster in context-first trials than in context-last trials, *b* = 0.19, 95% CI [0.09, 0.29], *t*(46.8) = 3.77, *p* < 0.001. This difference was larger in the selective-2 condition relative to both selective-1, *b* = 0.34, 95% CI [0.27, 0.42], *t*(9444.1) = 8.59, *p* < 0.001, and global conditions, *b* = −0.95, 95% CI [−1.02, −0.87], *t*(9444.3) = −23.61, *p* < 0.001. We observed significant RT benefits for context-first trials only in the selective-1 (mean difference = −153 ms, 95% CI [−241, −64], *t*(70.4) = −4.92, *p* < 0.001) and selective-2 conditions (mean difference = −345 ms, 95% CI [−434, −256], *t*(71.1) = −11.08, *p* < 0.001). In contrast, in the global condition, responses were faster on context-last trials than on context-first trials (mean difference = 182 ms, 95% CI [93, 270], *t*(70.0) = −4.08, *p* < 0.001; Figure 3A and Tables 1, 2). Additionally, in the context-first condition, RTs increased with the relevant set size, with significantly slower responses in the global compared to the selective conditions (mean difference = 187 and 151 ms, 95% CI [123, 251] and [85, 217], *t*(83.3) = 8.31 and *t*(80.1) = 5.27, *p*s < 0.001). Of central interest, however, was how performance in the context-last condition varied depending on relevant set size: Response latencies were shorter when one item was relevant (selective-1 context-last) compared to when two items were relevant (selective-2 context-last; mean difference = 228 ms, 95% CI [148, 308], *t*(69.9) = 8.15, *p* < 0.001). In contrast, globally retrieving all three maintained items was associated with faster responses than both selective context-last conditions (mean difference = −148 and − 376 ms, 95% CI [−212, −84] and [−442, −309], *t*(85.4) = −6.54 and *t*(82.3) = −16.01, *p*s < 0.001).

**Table 1:** Estimated parameters of response time models for readiness prompt (Experiment 1).

Fixed effects	Standardized coefficient	*df*	*t*-value	*p*-value	95% CI
Context position	0.19	46.8	3.77	<0.001	[0.09, 0.29]
Relevant set size (selective-2 vs. 1)	0.24	46.4	5.23	0.32	[0.15, 0.33]
Relevant set size (global vs. selective-2)	−0.20	46.5	−5.53	<0.001	[−0.27, −0.13]
Context position × selective-2 vs. 1	0.34	9444.1	8.59	<0.001	[0.27, 0.42]
Context position × global vs. selective-2	−0.95	9444.3	−23.61	<0.001	[−1.02, −0.87]

CI, confidence interval.

**Table 2:** *Post-hoc* analysis of response times to the readiness prompt (Experiment 1).

Contrast	Condition	Estimated mean difference	*df*	*t*-value	*p*-value	95% CI
Context-first vs. context-last	Selective-1	−153	70.4	−4.92	<0.001	[−241, −64]
	Selective-2	−345	71.1	−11.08	<0.001	[−434, −256]
	Global	182	70.0	4.08	<0.001	[93, 270]
Selective-2 vs. 1	Context-first	36	67.5	1.31	0.86	[−43, 115]
	Context-last	228	69.9	8.15	<0.001	[148, 308]
Global vs. selective-2	Context-first	151	80.1	5.27	<0.001	[85, 217]
	Context-last	−376	82.3	−16.01	<0.001	[−442, −309]
Global vs. selective-1	Context-first	187	83.3	8.31	<0.001	[123, 251]
	Context-last	−148	85.4	−6.54	<0.001	[−212, −84]

CI, confidence interval.

#### Probe response accuracy

Overall, response accuracy in the recognition test was very high. As expected, performance was better in context-first trials than in context-last trials, *b* = −0.65, 95% CI [−0.86, −0.45], *z* = −6.29, *p* < 0.001, and for non-intrusion compared to intrusion probes, *b* = −1.01, 95% CI [−1.26, −0.76], *z* = −7.89, *p* < 0.001 (Figure 3B and Tables 3, 4). The latter effect was most pronounced in the selective-2 context-last condition, *b* = −0.99, 95% CI [−1.96, −0.01], *z* = −1.99, *p* = 0.047. Error rates were higher in selective-2 context-last trials than selective-1 context-last trials, but only for intrusion probes (odds ratio = 2.36, 95% CI [1.41, 3.93], *z* = 4.90, *p* < 0.001), while no performance differences were observed for non-intrusion probes (odds ratio = 0.88, 95% CI [0.44, 1.77], *z* = −0.52, *p* = 1.00). Moreover, accuracy did not differ between the global condition (which included only non-intrusion probes) and selective-2 trials with non-intrusion probes (odds ratio = 0.93, 95% CI [0.51, 1.71], *z* = −0.35, *p* = 1.00). Relevant set size had no effect on accuracy rates in context-first trials (odds ratio = 1.18 and 1.18, 95% CI [0.45, 3.18] and [0.61, 2.25], *z* = 0.50 and 0.73, *p* = 1.00 and 1.00).

**Table 3:** Estimated parameters of the response accuracy models for the recognition test.

Fixed effects	Standardized coefficient	*z*-value	*p*-value	95% CI
Context position	−0.65	−6.29	<0.001	[−0.86, −0.45]
Relevant set size (Selective-2 vs. 1)	−0.26	−2.14	0.032	[−0.51, −0.02]
Relevant set size (Global vs. selective-2)^a^	−0.34	−2.06	0.040	[−0.66, −0.02]
Probe type (Intrusion vs. non-intrusion)^b^	−1.01	−7.89	<0.001	[−1.26, −0.76]
Context position × selective-2 vs. 1	−0.20	−0.82	0.42	[−0.69, 0.28]
Context position × global vs. selective-2^a^	0.53	1.62	0.10	[−0.11, 1.18]
Context position × probe type^c^	−0.13	0.08	0.93	[−0.66, 0.72]
Selective-2 vs. 1 × probe type	−0.49	−1.97	0.049	[−0.97, 0.00]
Context position × selective-2 vs. 1 × probe type	−0.99	−1.99	0.047	[−1.96, −0.01]

CI, confidence interval. ^a^This contrast includes only trials with non-intrusion probes. ^b^Only selective-1 and selective-2 conditions included same-trial lures (intrusion probes). In the global condition, probes contained only other-trial lures (non-intrusion probes). ^c^This contrast includes only trials from the selective-1 and selective-2 conditions.

**Table 4:** *Post-hoc* analysis of recognition test response accuracy.

Contrast	Condition	Odds Ratio	*z*-value	*p*-value	95% CI
Context-first vs. context-last	*Non-intrusion*				
	Selective-1	2.61	3.25	0.017	[1.10, 6.21]
	Selective-2	1.96	2.39	0.23	[0.86, 4.45]
	Global	1.15	0.82	1.00	[0.70, 1.89]
	*Intrusion*^a^				
	Selective-1	1.54	2.02	0.49	[0.82, 2.86]
	Selective-2	3.08	6.04	<0.001	[1.79, 5.32]
Selective-2 vs. selective-1	*Context-First*				
	Non-Intrusion	1.18	0.50	1.00	[0.45, 3.18]
	Intrusion	1.18	0.73	1.00	[0.61, 2.25]
	*Context-Last*				
	Non-Intrusion	0.88	−0.52	1.00	[0.44, 1.77]
	Intrusion	2.36	4.90	<0.001	[1.41, 3.93]
Global vs. selective-2^b^	Context-First	0.55	−2.37	0.24	[0.26, 1.15]
	Context-Last	0.93	−0.35	1.00	[0.51, 1.71]
Intrusion vs. non-intrusion^a^	*Context-First*				
	Selective-1	0.39	−3.23	0.018	[0.16, 0.92]
	Selective-2	0.39	−3.50	0.007	[0.18, 0.86]
	*Context-Last*				
	Selective-1	0.66	−1.95	0.55	[0.35, 1.24]
	Selective-2	0.25	−6.93	<0.001	[0.14, 0.45]

CI, confidence interval. ^a^Only selective-1 and selective-2 conditions included same-trial lures (intrusion probes). In the global condition, probes contained only other-trial lures (non-intrusion probes). ^b^This contrast includes only trials with non-intrusion probes.

#### Probe RT

The effect of context-position on probe RTs varied between selective-1 and selective-2 trials, *b* = 0.19, 95% CI [0.09, 0.29], *t*(46.8) = 3.77, *p* < 0.001, as well as between selective-2 and global trials, *b* = 0.19, 95% CI [0.09, 0.29], *t*(46.8) = 3.77, *p* < 0.001 (Figure 3B and Tables 5, 6). In the selective-1 condition, there were no significant RT differences between context-last and context-first trials (mean difference = −16 ms, 95% CI [−37, 5], *t*(193.7) = 2.12, *p* = 0.22). In the global condition, participants responded faster on context-last than on context-first trials (mean difference = 35 ms, 95% CI [14, 56], *t*(191.0) = 4.54, *p* < 0.001). Only in the selective-2 condition were probe RTs slightly slower on context-last compared to context-first trials (mean difference = −45 ms, 95% CI [−66, −24], *t*(199.9) = −5.79, *p* < 0.001). As anticipated, probe RTs increased as the number of relevant items increased, from selective-1 to selective-2 trials, *b* = 0.59, 95% CI [0.55, 0.63], *t*(9535.9) = 29.45, *p* < 0.001, and from selective-2 to global trials, *b* = 0.28, 95% CI [0.23, 0.33], *t*(9535.0) = 11.47, *p* < 0.001. Notably, this effect was consistent across context-first and context-last trials, despite the opposing RT trends observed in selective-2 condition (slowing from context-first to context-last), *b* = 0.12, 95% CI [0.04, 0.20], *t*(9536.3) = 3.02, *p* = 0.003, and the global condition (acceleration from context-first to context-last), *b* = −0.30, 95% CI [−0.39, −0.20], *t*(9534.5) = −6.13, *p* < 0.001. Unlike accuracy rates, RTs did not differ between intrusion and non-intrusion probes, *b* = −0.01, 95% CI [−0.05, 0.04], *t*(9536.4) = −0.24, *p* = 0.81.

**Table 5:** Estimated parameters of the response times models for the recognition test.

Fixed effects	Standardized coefficient	*df*	*t*-value	*p*-value	95% CI
Context position	0.05	63.9	2.04	0.046	[0.01, 0.08]
Relevant set size (selective-2 vs. 1)	0.59	9535.9	29.45	<0.001	[0.55, 0.63]
Relevant set size (global vs. selective-2)^a^	0.28	9535.0	11.47	<0.001	[0.23, 0.33]
Probe type (intrusion vs. no intrusion)^b^	−0.01	9536.4	−0.24	0.81	[−0.05, 0.04]
Context position × selective-2 vs. 1	0.12	9536.3	3.02	0.003	[0.04, 0.20]
Context position × global vs. selective-2^a^	−0.30	9534.5	−6.13	<0.001	[−0.39, −0.20]
Context position × probe type^b^	0.06	9540.8	1.46	0.15	[−0.02, 0.15]
Selective-2 vs. 1 × probe type	0.04	9536.4	0.93	0.35	[−0.04, 0.12]
Context position × selective-2 vs. 1 × Probe type	0.06	9535.3	0.73	0.47	[−0.10, 0.22]

CI, confidence interval. ^a^This contrast includes only trials with non-intrusion probes. ^b^This contrast includes only the selective-1 and selective-2 conditions.

**Table 6:** *Post-hoc* analysis of recognition test response times.

Contrast	Condition	Estimated mean difference	*df*	*t*-value	*p*-value	95% CI
Context-first vs. context-last	Selective-1	−16	193.7	−2.12	0.22	[−37, 5]
	Selective-2	−45	199.9	−5.79	<0.001	[−66, −24]
	Global	35	191.0	4.54	<0.001	[14, 56]
Selective-2 vs. 1	Context-First	126	9544.2	18.93	<0.001	[108, 143]
	Context-Last	154	9545.5	22.66	<0.001	[136, 173]
Global vs. selective-2^a^	Context-first	101	9543.9	12.54	<0.001	[79, 123]
	Context-last	31	9543.3	3.74	0.001	[9, 53]

CI, confidence interval. ^a^This contrast includes only trials with non-intrusion probes.

### Discussion

Experiments 1 yielded two key findings. First, speed of WM access, as indicated by responses times to the readiness prompt, was significantly slower when two items had to be selected compared to when one item was relevant. Relatedly, the subsequent recognition test revealed that selecting two items was more error-prone than selecting one item, but this effect occurred only when intrusion probes required participants to differentiate between items within the same memory set. The performance differences between the selective-1 and selective-2 context-last conditions challenge the hypothesis that inhibition demands significantly slow down selective WM access. If inhibitory activity increased with the number of irrelevant items, RTs would be expected to slow more when two out of three items were irrelevant (selective-1) than when only one was irrelevant (selective-2). Instead, the findings suggest that prioritization primarily relied on the selective enhancement of relevant WM contents, a process that becomes progressively more time-consuming as the number of items requiring amplification over their competitors increases.

The second relevant finding of Experiment 1 was the replication of a clear RT benefit for global, non-selective access to all current WM contents, previously observed in studies with memory sets of only two items (Chatham et al., 2014; Unger et al., 2016). Participants responded more quickly to the readiness prompt when all three items were relevant compared to when only one or two items were relevant. This advantage did not carry over to the subsequent recognition test. Instead, RTs for classifying probes increased with the number of relevant items while accuracy rates did not differ between global and selective conditions. Thus, the performance gap between selective and global WM output gating found in previous studies appears largely driven by the need to distinguish relevant from irrelevant memory contents during the selection process, rather than by processing of the memory probe (cf. Shepherdson et al., 2018).^3^

The performance cost observed for dual-item vs. single-item access suggests that selective retrieval of multiple WM contents is competitive rather than fully parallel and unrestricted. This finding, however, contradicts previous studies using the dual-access paradigm (Oberauer and Bialkova, 2009; Oberauer, 2013). Arguably, several uncontrolled factors may have contributed to the differences between the selective-1 and selective-2 context-last conditions in Experiment 1. For example, the double-star cue displays for dual-item access were more visually complex and potentially harder to discriminate than the single-star cues, which also more closely resembled the format of the digits at encoding (presented one at a time in a single spatial location). Additionally, it is possible that the readiness RTs were influenced by subvocal rehearsal or metacognitive processes, such as post-retrieval monitoring. Crucially, the dual-access paradigm is designed to avoid such confounds by keeping cues and response requirements identical across conditions. Nonetheless, the original version did not account for temporal context effects, which may have biased earlier interpretations. To more conclusively address the question of serial vs. parallel access, Experiments 2a and 2b employed a modified version of the dual-access paradigm specifically designed to minimize these temporal effects.

## Experiments 2a and 2b

We made two changes to the original dual-access paradigm. First, rather than repeating items in pairs—which could lead to their binding into the same episodic memory trace—we repeated only one item at a time in two separate single-access tests and then assessed the cumulative repetition benefits in a subsequent dual-access test. Note that due to the sequential nature of the tests, the two repeated items were neither never used at the same time, making it also unlikely that they were merged into a unified ad-hoc chunk. Second, we included a condition in which the same target was repeated twice, rather than repeating two distinct items once. This manipulation was designed to test whether repeating a single target would yield greater benefits in the dual-access test when that target could not become temporally associated with a repeated non-target. Crucially, under fully parallel, cost-free retrieval, any increase in access speed for a single repeated target should not affect the overadditive benefit of repeating both targets.

**Figure 4:**
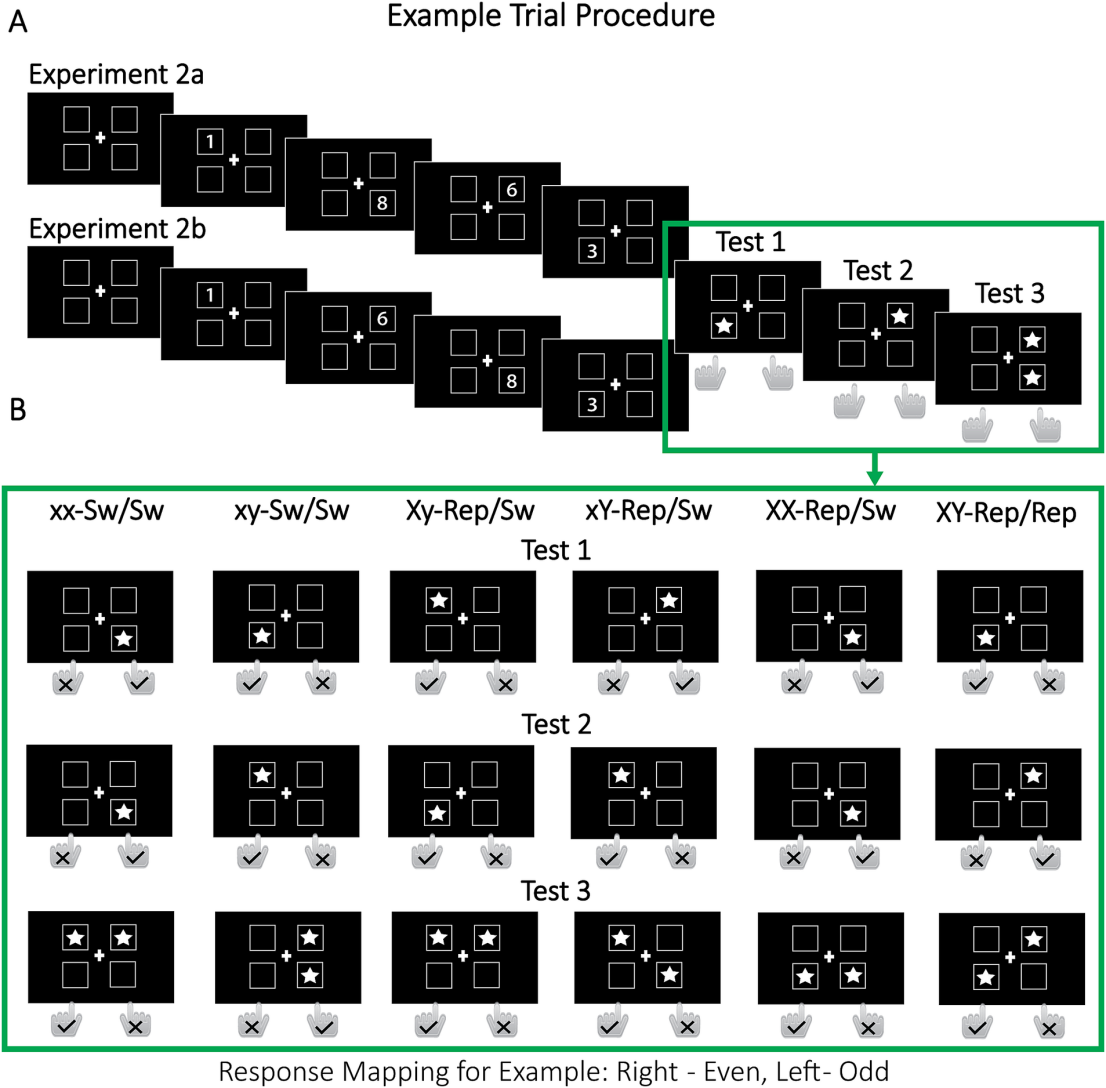
Example trial procedure for Experiments 2a and 2b. In Experiment 2a, four digits (1–9) were presented one at a time, each appearing in one of four locations chosen in random order. In Experiment 2b, the digits were shown in a fixed clockwise sequence starting from the upper left and proceeding to the lower left l. In Test 1, a star appeared in a randomly chosen location, and participants identified whether the digit at that location was odd or even using designated keys (e.g., “F” for odd, “J” for even). In test 2, a second star appeared in a randomly chosen location, which could be the same or different from the location in Test 1, prompting another odd/even decision. Finally, in Test 3, two stars appeared simultaneously in two randomly chosen locations, and participants determined whether the sum of the digits in those locations was odd or even. For instance, in the Xy-Repeat/Switch condition, in Test 1, the digit “1” was cued (odd, left key “F”). In Test 2, the digit “3” was cued (odd, left key “F”). In Test 3, the digits “1” (repeated target from Test 1) and “6” (non-repeated target) were cued, with their sum being 7 (odd, left key “F”). Sw, switch; Rep, repeat.

Experiments 2a and 2b followed similar procedures, with participants maintaining four digits (1–9) along with their spatial locations and/or sequential order in WM. The digits appeared in one of four squares, with spatial location serving as the retrieval cue (Figure 4A). In Experiment 2a, the sequential order of the four locations varied across trials to avoid a direct match between spatial location and serial position. Such spatial–temporal correspondence could lead to item access relying on the serial order of encoding, which may engage different mechanisms than retrieval in random order (Lange et al., 2011). In contrast, Experiment 2b used a fixed location order, mirroring the original dual-access paradigm. Fixing location order also addressed a practical issue: the randomized locations in Experiment 2a increased task difficulty, leading to comparatively high exclusion rates. Experiment 2b ensured that performance-based exclusion did not bias results. After encoding the digits, participants completed three cued memory tests. In the Tests 1 and 2, a single-star cue prompted them to decide whether the cued digit was odd or even (single-item access). In Test 3, a two-star cue prompted participants to indicate whether the sum of the cued digits was odd or even (dual-item access). The critical manipulation in Test 3 was whether each target had been previously probed in Test 1 and/or Test 2: it was possible that (i) neither of the two targets had been probed (switch/switch), (ii) one target had been probed once (single-repeat/switch), (iii) one target had been probed twice, i.e., in tests 1 and 2 (double-repeat/switch), or (iv) both targets had been probed (repeat/repeat; see Figure 4).

Our *predictions* for Experiments 2a and 2b hinge on the nature of dual-item retrieval: (1) If dual access is *serial*, then faster selection of any target should directly reduce overall RT. In this case, repeating one target (single-repeat/switch) should yield faster responses than no repetition (switch/switch), with an even greater benefit when the same target item is repeated twice (double-repeat/switch). Moreover, serial retrieval predicts that repeating both targets once (repeat/repeat) will produce an RT benefit roughly equal to the sum of the benefits from repeating each target individually (single-repeat/switch)—that is, an additive effect. A similar additive pattern is expected under *limited parallel* retrieval (cf. Figure 1). (2) In contrast, if dual access is *fully parallel* and *cost-free*, repeating only one target should confer minimal gains, regardless of whether that target is repeated once or twice. Here, a substantial RT reduction would occur only when both targets are repeated, resulting in an overadditive effect relative to conditions in which a single target is repeated. (3) Finally, we considered that, despite the temporal separation of the single-access tests, repeated items might still become sequentially associated (Lange et al., 2011). As noted earlier, such associations would yield disproportionally large benefits when both repeated items serve as targets in the dual-access test, but could be detrimental when one repeated item is a non-target. Thus, even if dual-item access is *not* fully parallel and cost-free, temporal context effects may mask the benefits of repeating a single target. Critically, repeating a single target twice (double-repeat/switch condition) provides an unbiased control because it precludes any temporal associations with another repeated item. Consequently, if the repeat/repeat condition yields an additive or underadditive benefit relative to the double-repat/switch condition, it would indicate that dual-item retrieval occur in a serial or limited parallel manner—even if temporal associations produce an overadditive benefit compared to the single-repeat/switch condition.

### Methods

#### Participants

One-hundred twenty-seven and 101 young adults participated in Experiments 2a and 2b, respectively. In Experiment 2a, there was some uncertainty in the power analysis due to differences in the response procedure and the effects of interest compared to Experiment 1. To account for this uncertainty, we aimed for a larger effective sample size of approximately *n* = 80, which was expected to provide sufficient to detect small to moderate RT differences between the individual conditions. For Experiment 2b, the target sample size (*n* = 60) was determined through power analyses based on the RT data and contrasts of interest from Experiment 2a, using the same power estimation methods as in Experiment 1. Participants were recruited from a research subject pool at Queens College, City University of New York (Experiment 2a: *n* = 49, Experiment 2b: *n* = 57), and from the crowdsourcing platform Prolific^©^ (Experiment 2a: *n* = 78, Experiment 2b: *n* = 44). Students at Queens College received course credit for their participation, while other participants were compensated with $12. All participants were pre-screened for age, mother tongue, and health. Only individuals who were between 18 and 40 years old, native English speakers, reported normal or corrected-to-normal vision and hearing, and were located in the United States took part in the studies. In Experiment 2a, 51 participants were excluded from data analysis due to insufficient trial numbers (fewer than 8 trials in any condition; see “Data Analysis” for details), including 31 participants from Prolific. Following the same criteria, 24 participants were excluded in Experiment 2b (13 participants from Prolific). The final sample for Experiment 2a included 76 datasets, the mean age was 25.1 years (*SD* = 5.81), ranging from 18 to 40 years. Thirty-five participants were female, 40 were male, one participant identified as non-binary/third gender (see Supplementary Methods for racial/ethnic information).

#### Materials, design, and procedure

##### Materials

Four randomly selected, unique digits (2.5° × 2.5°) were sequentially presented in one of four locations in the center of the screen (Figure 4A). The locations were marked by light-gray squares that extended over a visual angle of about 10.5° × 10.5°. Stars cued the locations of relevant items.

##### Design

The design included six within-subjects experimental conditions (Figure 4), determined by (i) the repetition status of the two target items in the dual-access test (repeated/unrepeated) and (ii) whether the same item or different items were probed in the two preceding single-access tests. Based on the repetition status, we defined three superordinate conditions: switch/switch, repeat/switch, and repeat/repeat. In the *switch/switch* condition, neither of the two target digits had been probed in the single-access tests. For example, given a memory set of {1, 3, 5, 6}, the cued digits across the three tests might be “5” (first single-access test 1), “1” (second single-access test), and “6” & “3” (dual-access test). In the *repeat/switch* condition, one of the two target digits had been probed earlier (e.g., “1” – “6” – “6” & “3”), whereas in the *repeat/repeat* condition, both target digits had been probed earlier (e.g., “6” – “3” – “6” & “3”). Each condition was further subdivided based on whether the same or different items were probed in the single-access tests. In the repeat/switch condition, we distinguished three cases: (i) *Xy-repeat/switch,*^4^ where the repeated target was accessed in Test 1 (e.g., “3” – “5” – “6” & “3”); (ii) *xY-repeat/switch*, where the repeated target was accessed in Test 2 (e.g., “1” – “6” – “6” & “3”); and (iii) *XX-repeat/switch*, where the repeated target was accessed in both tests (e.g., “3” – “3” – “6” & “3”). We expected repetition benefits to be stronger when a target had been accessed more recently (i.e., in Test 2), as its activation state would be heightened, facilitating retrieval more strongly. In contrast, probing the target in Test 1 and a non-target in Test 2 could make an irrelevant competitor more accessible. Additionally, the dual-access test (test 3) shared a more similar temporal context with Test 2 than with Test 1, making this context a more effective retrieval cue for the digit probed in Test 2. In the switch/switch condition, we further distinguished between *xx-switch/switch* trials in which the same item was probed in both single-access tests (e.g., “5” – “5” – “6” & “3”), and *xy-switch/switch* trials in which different items were probed (e.g., “5” – “1” – “6” & “3”). Accessing the same item twice (xx-switch/switch, xx-repeat/switch) was expected to create a stronger retrieval bias, making it more difficult to retrieve the non-repeated targets (in this case, “3” and “6”; Bäuml and Kliegel, 2017). This bias should be less pronounced when different items are cued in Tests 1 and 2, as in the xy-switch/switch and Xy/xY-repeat/switch conditions. To minimize the influence of such biases on repetition benefit estimates, we used xy-switch/switch trials as the baseline for assessing the repetition benefits in the Xy/xY-repeat/switch conditions, while xx-switch/switch trials served as the baseline for assessing repetition benefits in the XX-repeat/switch condition. The two dependent variables were accuracy and RT in dual-access test. The main focus was on response latencies.

##### Procedure

Before starting the experimental task, participants completed 20 practice trials, in which they had to reach a performance criterion of at least 60% correct answers across all 60 decisions (20 trials × 3 memory tests). Each trial began with the sequential presentation of four unique digits (1–9) in one of four on-screen locations (Figure 4A). Digit were displayed for 500 ms each, separated by a 250 ms inter-stimulus interval. The four spatial locations, marked by squares that remained visible throughout the trial, were presented in randomized order (Experiment 2a) or fixed order (upper left, upper right, lower right, lower left; Experiment 2b). After encoding the stimulus set, participants completed three memory tests. The first and second tests required single-item access: a star highlighted one location, and participants indicated whether the corresponding digit was odd or even. The third test required dual-item access: two stars cued the locations of two target digits, and participants determined whether their sum was odd or even. Response were made using the “F” or “J” keys with the left and right index fingers, respectively, with key assignments counter-balanced across participants. Stars appeared for 500 ms at randomly selected locations, allowing the cued digits to repeat or switch unpredictably across the three tests. Each test was separated by 250 ms fixation interval, while trials were separated by a 500 ms interval. All fixation intervals displayed the location placeholders and a central fixation cross. The main task comprised six blocks of 40 trials each, resulting in a total of 240 trials. The entire session lasted about 60 min. All other procedures were identical to those in Experiment 1.

#### Data analysis

##### Data preprocessing

In a first step, trials were excluded if they met at least one of the following criteria: (i) they were the first trial in a block, or (ii) response latencies were under 200 ms or over 10 s for any of the three consecutive memory tests. In a second step, trials with RTs exceeding three standard deviations above the individual mean for each condition and each of the three sequential decisions (first, second, third) were removed. Only trials with correct responses to all three retro-cues were included in the RT analysis. Datasets of individual participants were discarded if the latter criterion led to fewer than eight trials in any of the experimental conditions. We chose two approaches to analyze the data.

##### Pairwise condition contrasts (accuracy and RT)

The first approach was to set up linear mixed-effects models for both RT and accuracy data that included the fixed effect *condition* (xx-switch/switch, xy-switch/switch, Xy-repeat/switch, xY-repeat/switch, XX-repeat/switch, and XY-repeat/repeat) as well as participant-wise random intercepts. Random slopes effects were not supported by the data and thus excluded from the analysis. To determine whether there were any performance differences between the experimental conditions, a full model that included five contrasts to code for the fixed condition effect was compared to a null model that included participant-specific random intercepts only. This analysis was followed up with a set of custom post-hoc comparisons which were implemented using the *emmeans* package (v1.10.1, Lenth, 2024). Those comparisons included pairwise contrasts for all hypothesized differences between the conditions as well as two contrast terms that modelled additive repetition benefits. Specifically, the contrasts were coded as (i) (xy-switch/switch – repeat/repeat) – 2*(xy-switch/switch – 0.5*(xY-repeat/switch + Xy-repeat/switch)) to test for an additive repetition benefit of repeating a single target once, and (ii) (xy-switch/switch – repeat/repeat) – 2*(xx-switch/switch –XX-repeat/switch) to test for an additive repetition benefit of repeating a single target twice. Šidák correction was used to control the family-wise error rate.

##### Direct comparison of the dual-access hypotheses (RT)

In the second analysis approach, which focused on the RT data only, we created three predictors reflecting the expected performance patterns under different dual-item access hypotheses: (i) unrestricted parallel access, (ii) serial/limited parallel access without temporally-based association of the repeated items, and (iii) serial/limited parallel access with temporally-based association of the repeated items (see Table 7, cf. Oberauer and Bialkova, 2009, for a similar strategy). The three predictors were included as fixed effects, along with a random intercept for participants. The weights estimated for each predictor indicated the extent to which its corresponding RT pattern matched the observed data, allowing for a direct comparison of the three hypotheses. To further evaluate the individual contribution of each predictor, we assessed whether its removal substantially reduced model fit. This was determined based on changes in log-likelihood using a likelihood ratio test (implemented in the *anova* function in R), as well as difference in the Akaike information criterion (AIC) and Bayes information criterion (BIC).

**Table 7:** Predictor coding (Experiments 2a and 2b).

Predictor	Condition					
	xy-sw/sw	xx-sw/sw	Xy-rep/sw	xY-rep/sw	XX-rep/sw	XY-rep/rep
Unrestricted parallel	1	1	1	1	0	1
Serial, no temporal association	1	1	0.5	0.5	0	0.5
Serial, temporal association	1	1	1	1	0	0.5

The expected RT pattern was coded as follows: 1 = slowest RT (no repetitions benefit), 0.5 = medium RT (moderate repetition benefit), 0 = fastest RT (large repetition benefit); sw, switch; rep, repeat.

### Results

#### Accuracy

**Figure 5:**
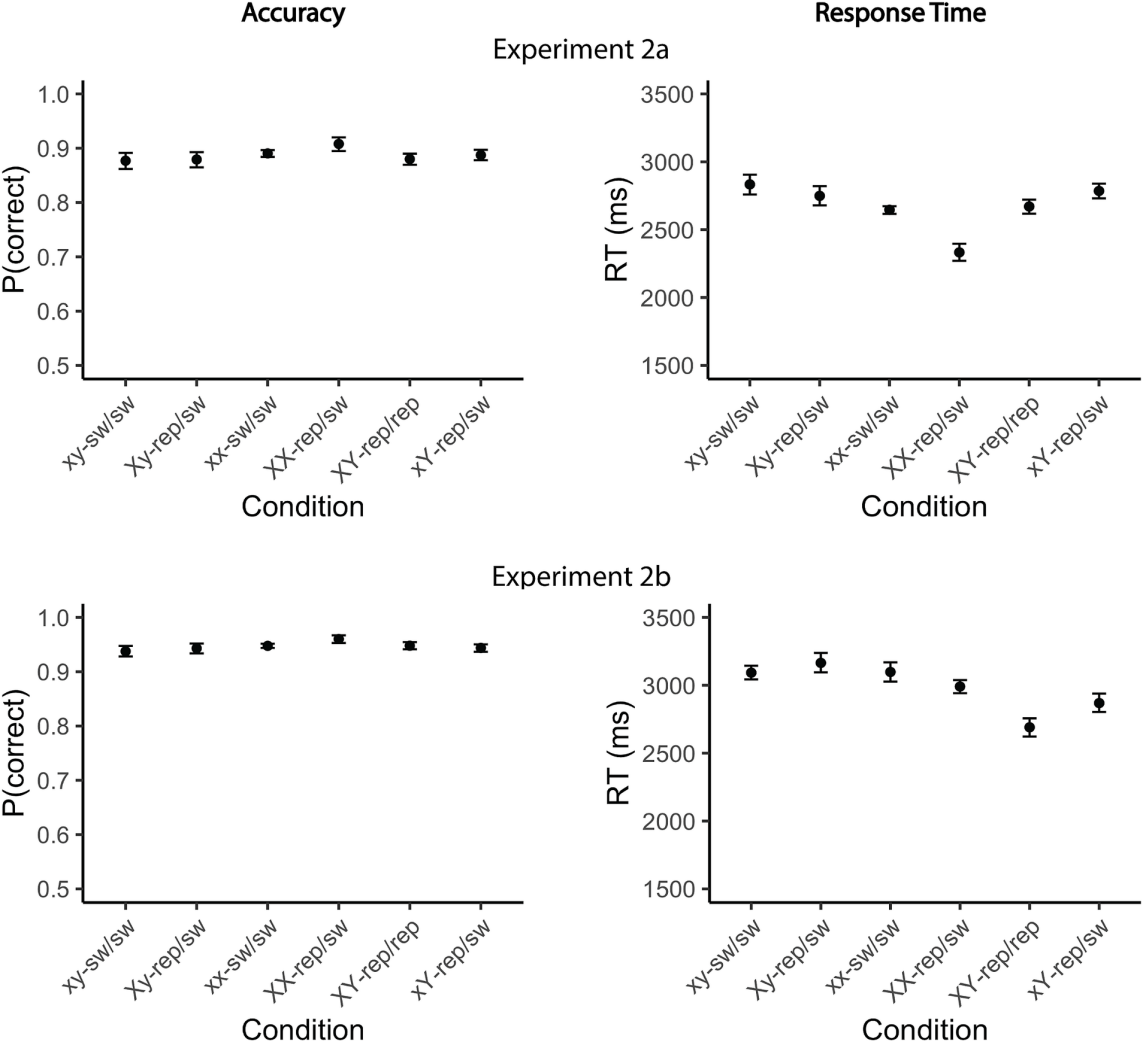
Mean accuracy rates and reaction times for Experiments 2a and 2b. Accuracy rates (left) and mean response time (right) in the dual-access condition (Test 3) are shown for the different conditions in Experiment 2a (top) and Experiment 2b (bottom). Conditions were categorized based on two factors: (1) Target repetition—neither target in Test 3 was repeated (xx-sw/sw, xy-sw/sw), one target was repeated (Xy-rep/sw, xY-rep/sw, XX-rep/sw), or both targets were repeated (XY-rep/rep); and (2) Item consistency across Tests 1 and 2—whether the same item was probed in both Test 1 and Test 2 (xx-sw/sw, XX-rep/sw), or different items were probed (xy-sw/sw, XY-rep/rep, Xy-rep/sw, xY-rep/sw). Upper-case letters in the condition names indicate digits that were targets in Test 3, while lower-case letters denote non-targets. For example, in the Xy-rep/sw condition, two different digits were probed in Tests 1 and 2. The digit in Test 1 (X) was a target in Test 3, whereas the digit in Test 2 (y) was a non-target in Test 3. Error bars represent within-subjects confidence intervals (95%), estimated using bootstrapping (percentile-based intervals). sw, switch; rep, repeat.

The analysis primarily focused on the RT data, with accuracy rates analyzed mainly to assess potential speed-accuracy trade-offs across the experimental conditions. As expected, overall response accuracy was higher Experiment 2b (93%) compared to Experiment 2a (85%; Figure 5, left panel). In *Experiment 2a*, participants committed fewer errors when a single target was repeated twice compared to when neither target was repeated (xx-switch/switch vs. XX-repeat/switch: odds ratio = 0.74, 95% CI [0.57, 0.95], *z* = −3.27, *p* = 0.008; xy-switch/switch vs. XX-repeat/switch: odds ratio = 0.75, 95% CI [0.58, 0.97], *z* = −3.07, *p* = 0.017). In contrast, no significant performance benefit was observed when a single target was probed only once, either in Test 1 (xy-switch/switch vs. Xy-repeat/switch: odds ratio = 0.92, 95% CI [0.74, 1.14], *z* = −1.03, *p* = 0.94) or Test 2 (xy-switch/switch vs. xY-repeat/switch: odds ratio = 0.99, 95% CI [0.80, 1.23], *z* = −0.15, *p* = 1.00). Furthermore, accuracy was higher when both targets were repeated compared to when a single target was repeated once (Xy-repeat/switch vs. repeat/repeat: odds ratio = 0.80, 95% CI [0.63, 1.00], *z* = −2.73, *p* = 0.049; xY-repeat/switch vs. repeat/repeat: odds ratio = 0.74, 95% CI [0.59, 0.93], *z* = −3.57, *p* = 0.003). In *Experiment 2b*, no reliable performance differences were found, except for slightly higher error rates in the Xy-repeat/switch condition compared to the repeat/repeat condition (odds ratio = 0.70, 95% CI [0.51, 0.95], *z* = −3.17, *p* = 0.012). All observed effects were small (Table 8).

**Table 8:** Accuracy model statistics and pairwise condition contrasts (Experiments 2a and 2b).

Contrast	Estimated odds ratio	*z*-value	*p*-value	95% CI
*Experiment 2a* Full model: AIC = 12,266, BIC = 12,320, *R^2^_adj_* = 0.233^a^ Intercept-only model: AIC = 12,283, BIC = 12,298, *R^2^_adj_* = 0.229^a^ Model comparison: *χ^2^(5)* = 26.47, *p* < 0.001				
xy-switch/switch vs. Xy-repeat/switch	0.92	−1.03	0.94	[0.74, 1.14]
xy-switch/switch vs. xY-repeat/switch	0.99	−0.15	1.00	[0.80, 1.23]
xx-switch/switch vs. XX-repeat/switch	0.74	−3.27	0.008	[0.57, 0.95]
xy-switch/switch vs. XX-repeat/switch	0.75	−3.07	0.017	[0.58, 0.97]
Xy-repeat/switch vs. xY-repeat/switch	1.07	1.07	0.93	[0.90, 1.28]
Xy-repeat/switch vs. repeat/repeat	0.80	−2.73	0.049	[0.63, 1.00]
xY-repeat/switch vs. repeat/repeat	0.74	−3.57	0.003	[0.59, 0.93]
XX-repeat/switch vs. repeat/repeat	0.98	−0.20	1.00	[0.75, 1.28]
*Experiment 2b* Full model: AIC = 8,073, BIC = 8,094, *R^2^_adj_* = 0.167^a^ Intercept-only model: AIC = 8,079, BIC = 8,127, *R^2^_adj_* = 0.171^a^ Model comparison: *χ^2^(5)* = 16.38, *p* < 0.001				
xy-switch/switch vs. Xy-repeat/switch	0.98	−0.16	1.00	[0.74, 1.31]
xy-switch/switch vs. xY-repeat/switch	0.92	−0.83	0.98	[0.69, 1.22]
XX-repeat/switch vs. xx-switch/switch	0.80	−1.87	0.40	[0.63, 1.23]
XX-repeat/switch vs. xy-switch/switch	0.88	−1.07	0.93	[0.63, 1.22]
Xy-repeat/switch vs. xY-repeat/switch	0.93	−0.83	0.98	[0.74, 1.18]
Xy-repeat/switch vs. repeat/repeat	0.70	−3.17	0.012	[0.51, 0.95]
xY-repeat/switch vs. repeat/repeat	0.75	−2.51	0.09	[0.55, 1.03]
XX-repeat/switch vs. repeat/repeat	0.78	−1.90	0.38	[0.55, 1.12]

CI, confidence interval. ^a^The small difference in R2 between the null model and the full model suggests that most of the variability in accuracy was due to mean interindvidual performance differences rather than intraindividual differences across the conditions.

#### RT

In both experiments, RTs varied across conditions, as indicated by a significant loss of fit when comparing the full model to the null model that contained only a random intercept [Experiment 2a: *χ*^2^(5) = 166.05, *p* < 0.001, ΔAIC = 156, ΔBIC = 119; Experiment 2b: *χ*^2^(5) = 139.50, *p* < 0.001, ΔAIC = 130, ΔBIC = 92].

##### Single-target repetition benefit

Pairwise comparisons revealed that repeating a single target *once* did not yield an RT benefit relative to the baseline condition, in which no items were repeated, regardless of whether the repeated target was probed in Test 1 (Xy-repeat/switch vs. xy-switch/switch; mean difference = −36 and 4 ms, 95% CI [−158, 86] and [−117, 125], *t*(10712.0) = −0.82 and *t*(12528.1) = 0.10, *p*s = 1.00) or Test 2 (xY-repeat/switch; mean difference = 80 and 108 ms, 95% CI [−43, 202] and [−13, 229], *t*(10711.4) = 1.83 and *t*(12528.0) = 2.50, *p*s = 0.50 and 0.12; Figure 5, right panel). However, RTs were significantly faster when the target had been probed more recently, i.e., in Test 2, than when it had been probed in Test 1 (mean difference = 116 and 103 ms, 95% CI [15, 217] and [5, 202], *t*(10711.3) = 3.22 and *t*(12527.9) = 2.95, *p*s = 0.013 and 0.031). Notably, respond speed also increased when a single target was repeated *twice* compared to when neither target was repeated (XX-repeat/switch vs. xx-switch/switch: mean difference = 327 and 296 ms, 95% CI [190, 464] and [159, 432], *t*(10711.5) = 6.67 and *t*(12527.5) = 6.08, *p*s < 0.001).

##### Dual-target repetition benefit

Responses were faster when both targets were repeated (repeat/repeat) compared to when only one target was repeated, regardless of whether the single target was repeated once (Xy-repeat/switch vs. repeat/repeat: mean difference = 454 and 401 ms, 95% CI [335, 573] and [283, 520], *t*(10711.2) = 10.66 and *t*(12527.3) = 9.51, *p*s < 0.001; xY-repeat/switch vs. repeat/repeat: mean difference = 338 and 298 ms, 95% CI [218, 458] and [180, 416], *t*(10711.2) = 7.90 and *t*(12527.7) = 7.05, *p*s < 0.001) or twice (mean difference = 116 and 103 ms, 95% CI [15, 217] and [5, 202], *t*(10711.3) = 3.22 and *t*(12527.9) = 2.95, *p*s = 0.013 and 0.031). Importantly, the RT benefit of the repeat/repeat condition was not uniform across the different repeat/switch conditions: The benefit was clearly overadditive compared to trials in which a single item was repeated once (mean difference = 374 and 294 ms, 95% CI [203, 545] and [124, 463], *t*(10711.7) = 6.12 and *t*(12527.2) = 4.86, *p*s < 0.001). However, it was numerically—though not statistically—underadditive relative to trials in which the item was repeated in both tests 1 and 2 (mean difference = −236 and − 185 ms, 95% CI [−543, 71] and [−120, 491], *t*(10711.3) = −2.15 and *t*(12528.1) = −1.70, *p*s = 0.27 and 61). The RT effects followed the same direction as the accuracy effects, making it unlikely that the findings reflect a speed-accuracy tradeoff (Table 9).

**Table 9:** Response time model statistics and pairwise condition contrasts (Experiments 2a and 2b).

Contrast	Estimated mean difference	*df*	*t*-value	*p*-value	95% CI
*Experiment 2a* Full model: AIC = 185,450, BIC = 185,509, *R^2^_adj_* = 0.48 Intercept-only model: AIC = 185,606, BIC = 185,628, *R^2^_adj_* = 0.47^a^ Model comparison: *χ*^2^(5) = 166.05, *p* < 0.001					
xy-switch/switch vs. Xy-repeat/switch	−36	10712.0	−0.82	1.00	[−158, 86]
xy-switch/switch vs. xY-repeat/switch	80	10711.4	1.83	0.50	[−43, 202]
XX-repeat/switch vs. xx-switch/switch	327	10711.5	6.67	<0.001	[190, 464]
XX-repeat/switch vs. xy-switch/switch	243	10711.4	4.93	<0.001	[105, 381]
Xy-repeat/switch vs. xY-repeat/switch	116	10711.3	3.22	0.013	[15, 217]
Xy-repeat/switch vs. repeat/repeat	454	10711.2	10.66	<0.001	[335, 573]
xY-repeat/switch vs. repeat/repeat	338	10711.2	7.90	<0.001	[218, 458]
XX-repeat/switch vs. repeat/repeat	175	10710.9	3.60	0.003	[39, 310]
Additive benefit for repeating single target once^b^	374	10711.7	6.12	<0.001	[203, 545]
Additive benefit for repeating single target twice^c^	−236	10711.3	−2.15	0.27	[−543, 71]
*Experiment 2b* Full model: AIC = 218,227, BIC = 218,287, *R^2^_adj_* = 0.39^a^ Intercept-only model: AIC = 218,357, BIC = 218,379, *R^2^_adj_* = 0.38^a^ Model comparison: *χ*^2^(5) = 139.50, *p* < 0.001					
xy-switch/switch vs. Xy-repeat/switch	4	12528.1	0.10	1.00	[−117, 125]
xy-switch/switch vs. xY-repeat/switch	108	12528.0	2.50	0.12	[−13, 229]
XX-repeat/switch vs. xx-switch/switch	296	12527.5	6.08	<0.001	[159, 432]
XX-repeat/switch vs. xy-switch/switch	227	12527.9	4.63	<0.001	[90, 364]
Xy-repeat/switch vs. xY-repeat/switch	103	12527.9	2.95	0.031	[5, 202]
Xy-repeat/switch vs. repeat/repeat	401	12527.3	9.51	<0.001	[283, 520]
xY-repeat/switch vs. repeat/repeat	298	12527.7	7.05	<0.001	[180, 416]
XX-repeat/switch vs. repeat/repeat	179	12527.5	3.70	0.002	[44, 314]
Additive benefit for repeating single target once^b^	294	12527.2	4.86	<0.001	[124, 463]
Additive benefit for repeating single target twice^c^	−185	12528.1	−1.70	0.61	[−120, 491]

CI, confidence interval. ^a^The small difference in R2 between the null model and the full model suggests that most of the RT variability was due to interindvidual differences in mean response speed, regardless of differences between the conditions. Although very modest relative to the random effects, the fixed effects indicate reliable performance benefits that were consistently observed across two independent and heterogenous samples. The size of those benefits does not diminish their significance for distinguishing the competing hypotheses concerning the dynamics of dual-item access. ^b^The contrast was coded as (xy-switch/switch – repeat/repeat) – 2*[(xy-switch/switch) – 0.5*(xY-repeat/switch + Xy-repeat/switch)]. The positive difference clearly shows that the benefit of repeating both targets was overadditive compared to repeating one target once. ^c^The contrast was coded as (xy-switch/switch – repeat/repeat) – 2*(xx-switch/switch –XX-repeat/switch). The negative difference suggests that the benefit of repeating both targets was numerically smaller than the sum of the benefits from repeating one target twice. However, the data did not provide sufficient evidence to reject the null hypothesis of additive benefits.

##### Direct comparison of the three dual-access hypotheses

Next, we directly compared the three predictors that modeled RTs under the assumption that retrieval is (1) unrestricted parallel, (2) serial without temporal chaining of repeated items, or (3) serial with temporal chaining. The predictor for serial access with temporal associations between the repeated items was the only one that predicted a substantial portion of variance [*χ*^2^(1) = 30.52 and 18.61, *p*s < 0.001, ΔAIC = 29 and 16, ΔBIC = 21 and 9; see Table 10]. Although the predictor for serial access without chaining was significantly different from zero as well, its removal did not result in a substantial loss of fit [the BIC was larger for the full model compared to the reduced model; *χ*^2^(1) = 4.23 and 8.45, *p*s = 0.040 and 0.004, ΔAIC = 2 and 6, ΔBIC = −5 and – 1].

**Table 10:** Response time model statistics for parametric predictors (Experiments 2a and 2b).

Contrast	Estimate	95% CI	*χ* ^2^	*df*	*p*-value	ΔAIC	ΔBIC
*Experiment 2a* Full model: AIC = 185,460, BIC = 185,504, *R^2^_adj_* = 0.48							
Unrestricted parallel	−90	[−254, 74]	1.15	1	0.28	−1	−8
Serial without temporal chunking	132	[6, 257]	4.23	1	0.040	2	−5
Serial with temporal chunking	425	[274, 576]	30.52	1	<0.001	29	21
*Experiment 2b* Full model: AIC = 218,235, BIC = 218,279, *R^2^_adj_* = 0.39							
Unrestricted parallel	−66	[−229, 97]	0.63	1	0.43	−2	−9
Serial without temporal chunking	184	[60, 308]	8.45	1	0.004	6	−1
Serial with temporal chunking	327	[179, 476]	18.61	1	<0.001	16	9

CI, confidence interval.

### Discussion

Experiments 2a and 2b demonstrated a clear performance benefit when both target items were repeated, whereas no such advantage emerged when a single target was repeated *once*. At first glance, this overadditive benefit seems to support previous accounts suggesting that dual-item access can be truly parallel and cost-free (Oberauer, 2013). Crucially, however, repeating the same target *twice*, resulted in a substantial boost in response speed, and to a lesser extent in accuracy, relative to both trials in which neither target was repeated and those in which a single target was repeated once. When directly contrasted with repeating a single target twice, the benefit of repeating both targets was *not* overadditive, questioning the notion of unrestricted parallel dual-item access. Instead, our findings are most consistent with an account in which dual-item retrieval occurs either serially or in limited parallel manner, with repeated items becoming directly or indirectly associated. This association enhances dual-access performance when both repeated items are targets but hinders it when one is a non-target. Importantly, this interpretation received the strongest support when directly compared to the alternative hypotheses—(1) fully parallel cost-free retrieval and (2) serial/limited parallel retrieval without temporal associations—in a single statistical model.

The formation of such associations may seem surprising given our attempt to temporally segregate repeated access to individual items. One might instead argue that the present pattern of findings reflects that the effect of repeating a single target once is too subtle to detect but becomes more robust when the same target is probed twice. However, this account is difficult to reconcile with the significant performance improvement observed when both targets were repeated, despite each being accessed only once in the first or second test. Our findings are also unlikely to be explained by ad-hoc chunking, where two items are merged into a single unit before retrieval, with repetitions benefits arising from carrying over that chunk across retrieval attempts (Oberauer and Bialkova, 2009). In the present study, such chunking was improbable, as the repeated items were retrieved individually in two separate single-access tests. The Test 2 target likely replaced the Test 1 target in the focus of attention rather than both being integrated. In support of this notion, RTs in the dual-access test were faster when the target was probed in the second test compared to the first, possibly reflecting a lingering advantage from its recent presence in the focus of attention (Oberauer and Hein, 2012). Thus, the most plausible interpretation is that the repeated items become linked, so that retrieving one cues the retrieval of the other. This link facilitates dual-item access when both items are targets but creates interference when one is a non-target.

## General discussion

Building on previous findings that dual-item access can be as efficient as single-item access, this study investigated whether multi-item retrieval benefits arise from reduced inhibitory demands, ad-hoc chunking of the relevant items, or truly parallel, cost-free retrieval. Experiment 1 assessed the role of inhibition in cue-based WM retrieval by comparing selective single-item and dual-item access using a pre- and retro-cuing paradigm. If selection were slowed by the need to deactivate irrelevant contents, single-item access should be slower and less accurate than dual-item access. Instead, we observed a clear performance cost for dual-item access, challenging both the proposed role of inhibitory process in multi-item access and the notion of fully parallel, unrestricted retrieval. Experiments 2a and 2b further tested whether overadditive repetition benefits in a dual-access paradigm—previously attributed to cost-free parallel retrieval or chunking—could instead result from temporal associations between repeated items. To minimize these associations, we (i) repeated only one item at a time instead of repeating item pairs and (ii) introduced a control condition in which a repeated target could not be linked to a non-target. Under this latter condition, the RT benefit of repeating both dual-access targets were no longer overadditive but merely additive. Consistent with Experiment 1, these findings suggest that dual-item access is unlikely to be truly parallel and cost-free.

### Interference slows multi-item working memory access

Experiment 1 demonstrated that accessing two items is slower and more error prone than selecting a single item from WM. Notably, the performance cost was diminished in the global condition, when all three items had to be retrieved. This finding suggests that facilitated access to multiple items, observed in prior studies with memory sets of two items (Chatham et al., 2014; Unger et al., 2016), reflects inherently lower demands of global WM access compared to selective access. The cost of selective WM access has been proposed to arise from the interplay of context-driven enhancement of relevant memory representations and inhibition of irrelevant ones (Chatham et al., 2014; Oberauer, 2018). Consequently, an increase in the number of irrelevant items could amplify inhibitory activity, potentially causing greater RT slowing. Contrary to this notion, the results of Experiment 1 showed that RTs decreased as the number of irrelevant items increased. While this finding does not rule out the possibility that inhibitory activity contributes to some degree of slowing in selective WM access, it clearly indicates that this kind of activity is not the primary driver of performance differences between single- and multi-item retrieval.

The results from Experiments 2a and 2b instead suggest that selective retrieval of multiple WM representations is slower because it does not occur in an unrestricted, parallel manner. Simultaneously selecting two items—especially if selection depends on two distinct context representations, as in this study—appears to induce substantial interference and may even force retrieval to proceed serially. This interpretation is consistent with well-known restrictions of control-dependent behavior which are thought to arise from the need to limit interference between different processes that share neural representations (cf., Musslick and Cohen, 2021). However, our findings contradict prior research indicating that dual-item access can be truly parallel and cost-free or that it benefits from chunking relevant items into a single unit (Oberauer and Bialkova, 2009; Risse and Oberauer, 2011; see also Oberauer, 2013). We propose that a key to explain this disparity lies in the role of associative links formed between items when they are accessed in close temporal proximity. Specifically, we believe that these associations provide an alternative interpretation for the overadditive benefit of repeating both targets in a dual-access paradigm, which has been taken as main argument for cost-free parallel retrieval.

One possible mechanism is that repeated items become directly linked through forward chaining—a process proposed to underlie memory for serial order and the benefits of cue-based WM access that relies on serial adjacency (Murdock, 1995; Lange et al., 2011). In this scenario, cue-triggered retrieval of one repeated item in the subsequent dual-access test can automatically prompt retrieval of its associated partner. When that partner happens to be a non-target, its retrieval interferes with cue-based access to the non-repeated target; conversely, when both repeated items are targets, their mutual association facilitates dual-item access. Moreover, if both repeated items are non-targets, the fact that neither is cued in the dual-access test reduces the potential for interference with the relevant items.

A related possibility is that repeated items become indirectly linked through their shared association with overlapping temporal contexts in the single-access tests (Tests 1 and 2). On this view, cue-based retrieval of an item in Test 1 or Test 2 leads to the formation of an episodic memory trace that integrates the item, its spatial cue, and the prevailing temporal context (Howard and Kahana, 2002; Karpicke et al., 2014). Because the temporal context of the dual-access test resembles that of the preceding single-access tests, it facilitates retrieval of the repeated items. Moreover, when a repeated item is a target in the dual-access test, the presentation of its spatial cue—and the item itself—can reactivate features of the episodic traces from Tests 1 and 2 that also feed activation to the second repeated item (Bäuml and Kliegl, 2017). The consequences of indirect associations between repeated items are therefore similar as for direct associations: If the second item is a target, its additional activation will enhance dual-item retrieval; if it is a non-target, however, its activation interferes with retrieval of the non-repeated target. For instance, if the items “3” – “4” – “6 & 3” were probed in Tests 1 through 3, the repeated non-target (“4”) would receive stronger activation from the temporal context in Test 3 than the non-repeated target (“6”), whose retrieval primarily relies on its spatial context cue. In addition, the spatial cue for the repeated target (“3”) may further reactivate the episodic trace containing the repeated non-target (“4”). In contrast, when both repeated items are non-targets, their spatial cues are absent during the dual-access test, reducing reactivation of the episodic event representations from Tests 1 and 2. This, in turn, limits the potential for unintentional amplification of the non-targets. Thus, both direct and indirect association mechanisms might explain why retrieval of one repeated item could inadvertently activate the other, despite the temporal separation of the individual tests.

### Global access to working memory is less constrained than selective access

Assuming that simultaneous access to multiple items in WM is heavily constrained raises the question of why these limitations do not apply in the same extent to global retrieval of all contents. One possible answer is that global access does not depend on the same kind of unique, item-specific context representations that guide selective access (Lewandowsky and Farrell, 2008; Oberauer, 2019; Oberauer and Lin, 2017, 2023). For instance, it has been suggested that global retrieval may rely on a single joint context representation, such as the overall trial context (Oberauer, 2019; Farrell, 2012). Bindings to a shared group context could be formed at a different hierarchical level than the unique item-context bindings, which would make them less susceptible to interference (Collins and Frank, 2013; Oberauer, 2018). Alternatively, the greater efficiency of global relative to selective WM access might stem from active maintenance of all items within a trial, effectively separating current memoranda from previously presented information that is no longer actively maintained (e.g., Davelaar et al., 2005; Ma et al., 2014; see also Miller et al., 2018). In an activation-based account of WM, differentiating among the currently maintained items becomes more demanding when it requires relative changes in the activation levels for relevant and irrelevant representations, as in selective access. Implementing such changes likely requires additional processing time.

An interesting question for future research will be whether selective, multi-item retrieval can occur in a more unrestricted parallel manner when the relevant items are bound to the same group context. Support for this notion comes from previous retro-cuing studies, in which groups of several items were associated with a single context. In these studies, participants were either presented with a valid group context cue before selecting a single memory item from the relevant group or no specific group was cued and the relevant item had to be selected from among all maintained contents (Oberauer, 2018; Heuer and Schubö, 2016; Souza and Oberauer, 2016). Although participants had to complete two selection steps—first by selecting the items in the relevant group, then by selecting the relevant item within this group based on its unique associated context—accuracy did not drop compared to the condition without group context cues. In contrast, in the present study, accuracy was reduced when participants selected two items, each based on its unique context, rather than only a single item. This discrepancy in findings suggests that (i) selecting multiple items causes much less interference if it is based on a single group context rather than multiple, item-specific context representations, and (ii) selection on the level of the group context does not interfere with selection on the item-specific level.

## Conclusion

The present findings indicate that retrieving multiple items from WM based on item-specific context representations is both slower and more error-prone compared to accessing a single item. The additional cost of multi-item retrieval does not primarily arise from balancing the enhancement of relevant items against the inhibition of irrelevant ones. Instead, the results are more consistent with the broader notion that multi-item access is heavily constrained by competitive interactions and interference among the retrieval candidates and the associated context representations, a principle shared by several models of selective WM access (e.g., Frank and Badre, 2012; Oberauer and Lin, 2023). For instance, interference-based models of cue-driven WM retrieval imply that simultaneous activation of multiple context cues amplifies mutual distortion of the corresponding item (or feature) representations (Oberauer and Lin, 2017). Similarly, neurobiologically-inspired models of WM access (Chatham and Badre, 2015) suggest that unrestricted parallel access would require multiple cortico-striatal loops to engage concurrently without crosstalk—an unlikely scenarios given their overlapping projections and highly interconnected nature (Haber, 2016; Pennartz et al., 2009). The need to mitigate maladaptive effects of such competitive dynamics may explain why multi-item WM access does not occur in a cost-free, parallel manner.

## Data Availability

The datasets presented in this study can be found in online repositories. The names of the repository/repositories and accession number(s) can be found at: https://osf.io/xharw/?view_only=94fdc2551b9b4da69a7fe13e41031460.

## Footnotes

^1^It is important to note that there are differing views on whether item-context bindings are temporary or enduring (cf. Beukers et al., 2021).

^2^While the relationship between episodic memory and WM lies beyond the scope of this study, it is widely recognized that long-term memory mechanisms play a significant role in supporting performance on WM tasks (e.g., Barbosa et al., 2020; Bartsch and Oberauer, 2023; Ngiam et al., 2019).

^3^Notably, the results of Experiment 1 also provide a manipulation check, confirming that participants processed both the context cue and the readiness prompt as intended. Specifically, the results indicate that they (i) proactively selected relevant items based on the pre-cue rather than deferring selection until the end of the trial, and (ii) did not disregard the readiness prompt (see Supplementary Discussion).

^4^In the condition names, uppercase letters (X, Y) denote targets in the dual-access test, while lowercase letters (x, y) denote non-targets.
