# Erratum: Constraints on multi-item working memory access: performance costs and retrieval dynamics

**DOI:** 10.3389/fpsyg.2025.1632138

**Published:** 2025-06-11

**Authors:** Frontiers Production Office

**Affiliations:** Frontiers Media SA, Lausanne, Switzerland

**Keywords:** multi-item working memory access, output gating, parallel vs. serial working memory retrieval, retrocuing, working memory

Due to a production error, several Greek symbols (“χ^2^” and “Δ”) were inadvertently changed to the Latin alphabet (“*c*^2^” and “D”).

The publisher apologizes for this mistake.

The original version of this article has been updated.

