# Supplementary material for: Constraints on multi-item working memory access: performance costs and retrieval dynamics
Source: Front Psychol. 2025 Apr 9;16:1558689. doi: 10.3389/fpsyg.2025.1558689 (PMC12014675; doi:10.3389/fpsyg.2025.1558689)
Supplement: Supplementary file 1 [file Data_Sheet_1.docx]

**S1 Supplementary Methods**

***Ethnic and Racial Information***

**Experiment 1.** Forty-nine percent of the participants self-identified as non-Hispanic White, 17% as non-Hispanic Asian, 15% as non-Hispanic Black, 4% as Hispanic White, and 4% as Hispanic Other-Race. Two participants self-identified as Asian and White, respectively, and preferred not to report their ethnicity. One participant identified as non-Hispanic but did not report their race. Another participant did not report either race or ethnicity.

**Experiments 2a and 2b.** Twenty-one percent of the participants self-identified as non-Hispanic White, 11% as non-Hispanic Asian, 14% as non-Hispanic Black, 8% as Hispanic White, 3% as Hispanic Other-Race, and 3% as non-Hispanic Other-Race. One participant self-identified as non-Hispanic Native Hawaiian or Other Pacific Islander. One participant self-identified as White but preferred not to provide their ethnicity. The final sample for Experiment 2b included 77 datasets, the mean age was 23.5 years (*SD* = 5.48), ranging from 18 to 35 years. Forty-six participants were female, 32 were male. Thirty percent of the participants self-identified as non-Hispanic White, 26% as non-Hispanic Asian, 14% as non-Hispanic Black, 10% as Hispanic Other-Race, 6% as Hispanic White, 3% as non-Hispanic Other-Race, and 3% as Hispanic Black. One participant self-identified as Hispanic American Indian or Alaska Native and one as non-Hispanic American Indian or Alaska Native. Three participants self-identified as Asian but preferred not to provide their ethnicity, another two chose not to give information about their race and self-identified as Hispanic.

**S2 Supplementary Discussion (Experiment 1)**

Notably, the results of Experiment 1 also provided a manipulation check, confirming that participants processed both the context cue and the readiness prompt as intended. Readiness RTs were faster and probe response accuracy higher for selective context-first trials compared to selective context-last trials. This suggests that participants proactively used the pre-cue to encode only relevant items, thereby reducing WM load in selective-1 and selective-2 trials. Moreover, as expected if participants retrieved the relevant items immediately upon the readiness prompt, response speed in the subsequent recognition test increased with relevant set size—responses were faster when the pool of retrieval candidates was reduced to one or two out of the three items. Finally, previous research has shown that when the probe appears simultaneously with a retro-cue, response speed is considerably slower in the context-last condition than in the context-first condition, reflecting the additional time required to retrieve items from WM (Chatham et al, 2014; Unger et al., 2016). In contrast, in the present study, probe RTs were not substantially affected by context order. Together, these findings indicate that participants actively prepared for the recognition test rather than passively waiting for the probe. Still, when two items had to be selected, this preparation was somewhat less efficient or consistent in the context-last condition compared to the context-first condition.
